# Qualitative evaluation of regioselectivity in the formation of di- and tri-6-*O*-tritylates of α-cyclodextrin

**DOI:** 10.3762/bjoc.11.168

**Published:** 2015-09-02

**Authors:** Keisuke Yoshikiyo, Yoshihisa Matsui, Tatsuyuki Yamamoto

**Affiliations:** 1 Faculty of Life and Environmental Science, Shimane University, 1060 Nishikawatsu, Matsue, Shimane, 690-8504, Japan

**Keywords:** ^1^H and ^13^C NMR spectroscopy, quantitative analysis, regioselectivity, tritylation, ultra-fast liquid chromatography (UFLC)

## Abstract

The quantitative analysis of reaction products showed that the reaction of 6^A^,6^D^-di-*O*-trityl-α-cyclodextrin (CD), AD-isomer, with trityl chloride in pyridine at 55 °C gave 6^A^,6^B^,6^E^-tri-*O*-trityl-α-CD, the amount of which was only 25% of that of simultaneously formed 6^A^,6^B^,6^D^-tri-*O*-trityl-α-CD. This indicates that the bulky trityl groups of glucopyranose-A and -D (Glu-A and -D, respectively) in the AD-isomer mainly retard the additional tritylation of the C(6)-OH of the adjacent glucopyranoses in a counter-clockwise direction (Glu-F and -C, respectively). ^1^H NMR spectra of the AD-isomer showed that the O(6)-H and C(6)-H signals of Glu-C and -F are shifted upfield due to the ring current of the trityl groups. Thus, it is concluded that the bulky trityl groups on Glu-A and Glu-D are oriented to Glu-F and Glu-C, respectively, and sterically retard additional tritylation on Glu-F and Glu-C. Similar steric hindrance was also observed in the additional tritylations of mono-6-*O*-trityl-α-CD, 6^A^,6^B^-di- and 6^A^,6^C^-di-*O*-trityl-α-CD’s.

## Introduction

**Scheme 1:**
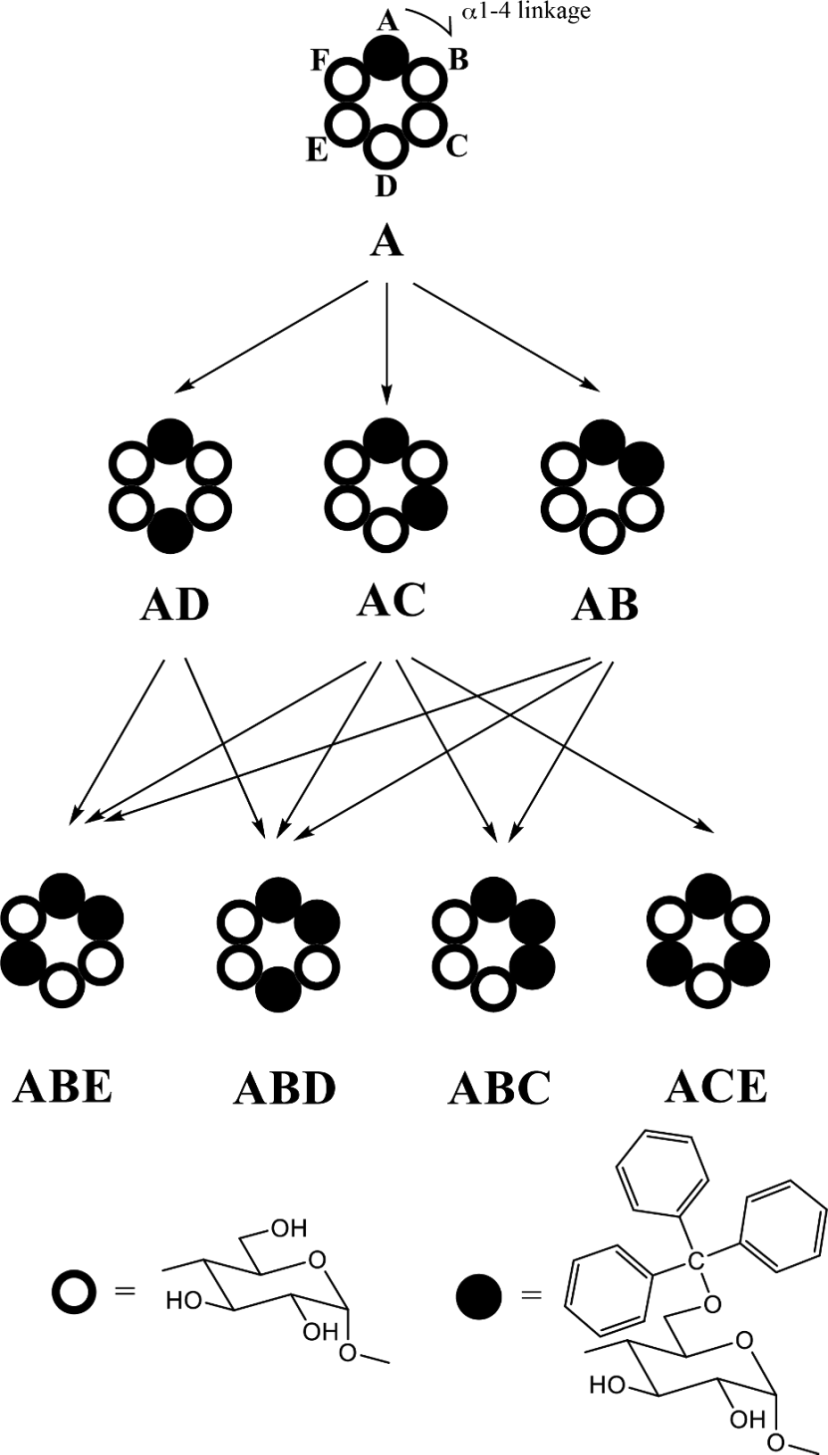
Tritylates of α-CD examined in the present study and the reaction pathway. Letters A to F represent the glucopyranose units along with α1-4 linkage direction, numbered when viewed from the secondary hydroxy group side.

Regioselective modification and deprotection on the primary hydroxy side of cyclodextrins (CDs) are of great importance in supramolecular chemistry, as they allow the preparation of sophisticated concave molecules such as multi- or hetero-functionalized CD derivatives that are important intermediates for the preparation of enzyme mimics [1–5]. The tritylation of cyclodextrin (CD) has attracted much attention, since the tritylates are useful intermediates for the preparation of a variety of functionalized CD derevatives [6–19]. CDs have hydroxy groups at the 2-, 3- and 6-positions of their glucopyranose (Glu) residues. However, the reaction of CD with trityl chloride in pyridine gives exclusively 6-*O*-substituted CD. CD is composed of six (α-CD), seven (β-CD), eight (γ-CD), or more Glu residues. Thus, a reaction of α-CD, for example, with trityl chloride (TrCl) gives mono- [7], di- [13,15–16], tri- [9,11], tetra- [18], and per-6-*O*-trityl derivatives [19]. The di-6-*O*-tritylate of α-CD involves three regioisomers, that are, 6^A^,6^B^-, 6^A^,6^C^-, and 6^A^,6^D^-di-*O*-tritylates (AB-, AC-, and AD-isomers, respectively), as shown in Scheme 1. These regioisomers were well separated and characterized [13]. The tri-6-*O*-tritylate of α-CD involves four regioisomers, that are 6^A^,6^B^,6^C^-, 6^A^,6^B^,6^D^-, 6^A^,6^B^,6^E^-, and 6^A^,6^C^,6^E^-tri-*O*-tritylates (ABC-, ABD-, ABE-, and ACE-isomers, respectively, Scheme 1). Among them, only a symmetrical molecule of the ACE-isomer was separated and characterized [9]. The tetratritylate of α-CD involves three regioisomers, among which 6^A^,6^B^,6^D^,6^E^-tetra-*O*-tritylate was separated and characterized [18]. The trityl group is so bulky that the additional tritylation of C(6)-OH on Glu adjacent to a previously tritylated Glu is considered to be sterically hindered. Thus, a reaction of α-CD with 3.3 equivalents TrCl in pyridine at 55 °C gave selectively the ACE-isomer in good yield (23%) [9]. However, the regioselectivity in the formation of di- or tri-6-*O*-trityl-α-CD has not always been quantitatively evaluated. The present work deals with the analysis of products in the reaction of α-CD, as well as mono- and di-6-*O*-trityl-α-CD’s, with TrCl in pyridine to evaluate quantitiatively the steric effect of the bulky trityl group on the regioselectivity of the reaction. For the sake of clarity, it must be noted here that letters from A to F that identify individual glucopyranose units are numbered along with the α1→4 linkage direction, and that the term “clockwise direction” refers to the direction when viewed from the secondary hydroxy side, as shown in Scheme 1.

## Results and Discussion

### Identification of di- and tri-6-*O*-tritylates of α-CD

**Figure 1:**
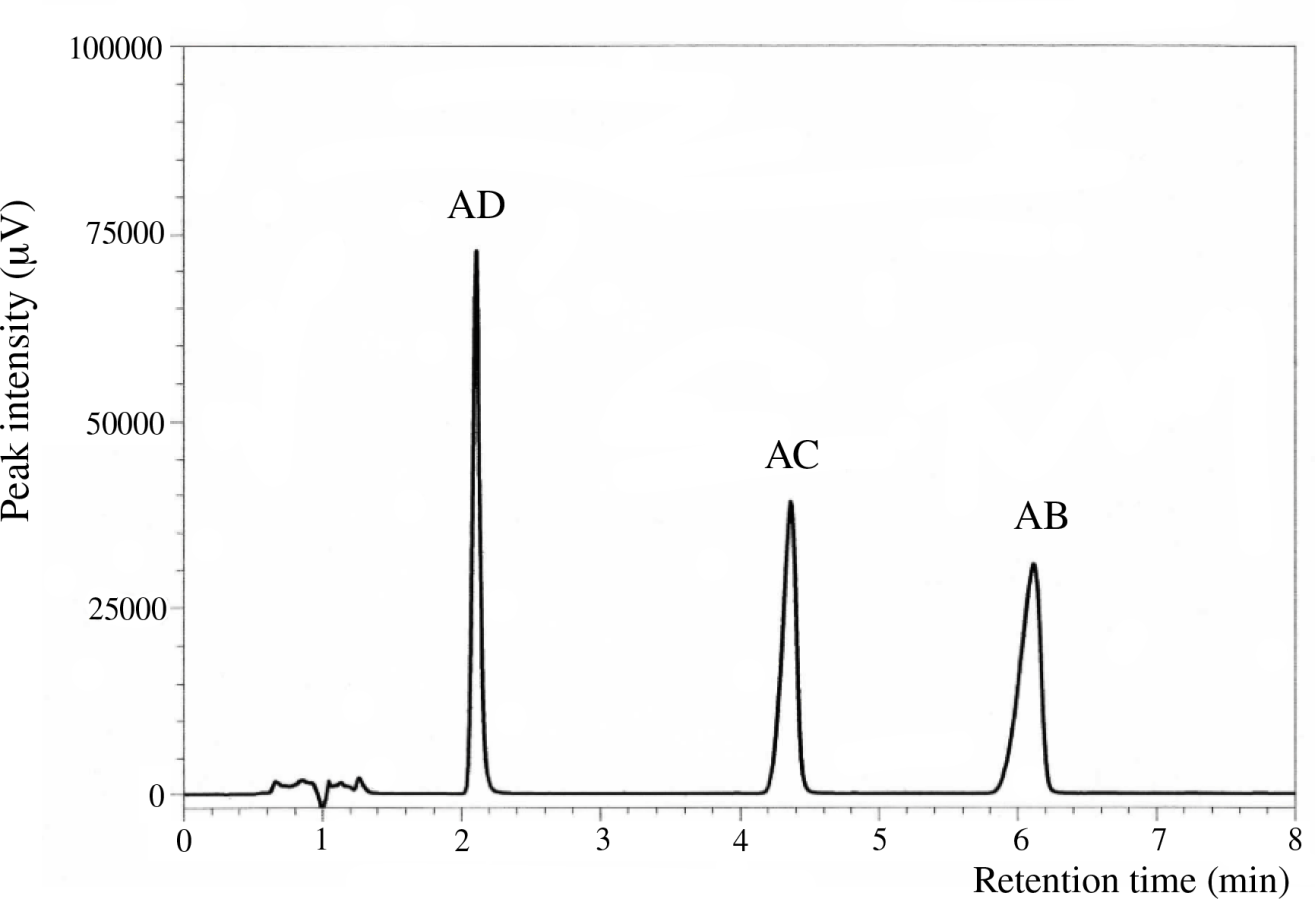
UFLC chromatogram of three regioismers of di-6-*O*-trityl-α-CD with 50% aqueous acetonitrile as an eluent.

**Figure 2:**
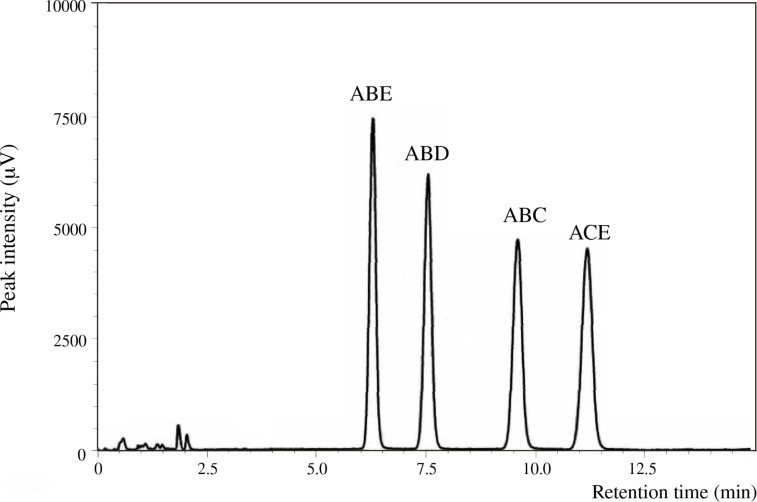
UFLC chromatogram of four regioisomers of tri-6-*O*-trityl-α-CD with an eluent of acetonitrile/methanol/water (40:45:15, v/v/v).

**Figure 3:**
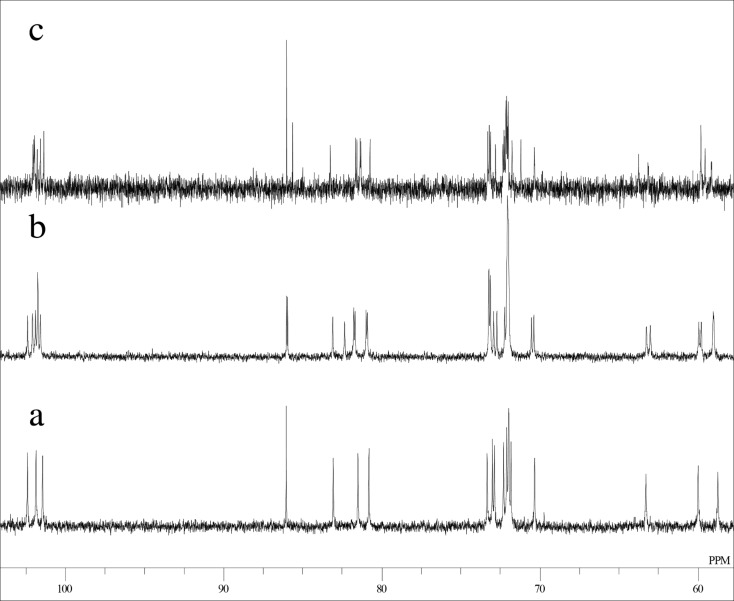
A part of ^13^C NMR spectra of di-6-*O*-trityl-α-CD in DMSO-*d*_6_ at 50 °C a) AD-isomer, b) AC-isomer, and c) AB-isomer.

**Scheme 2:**
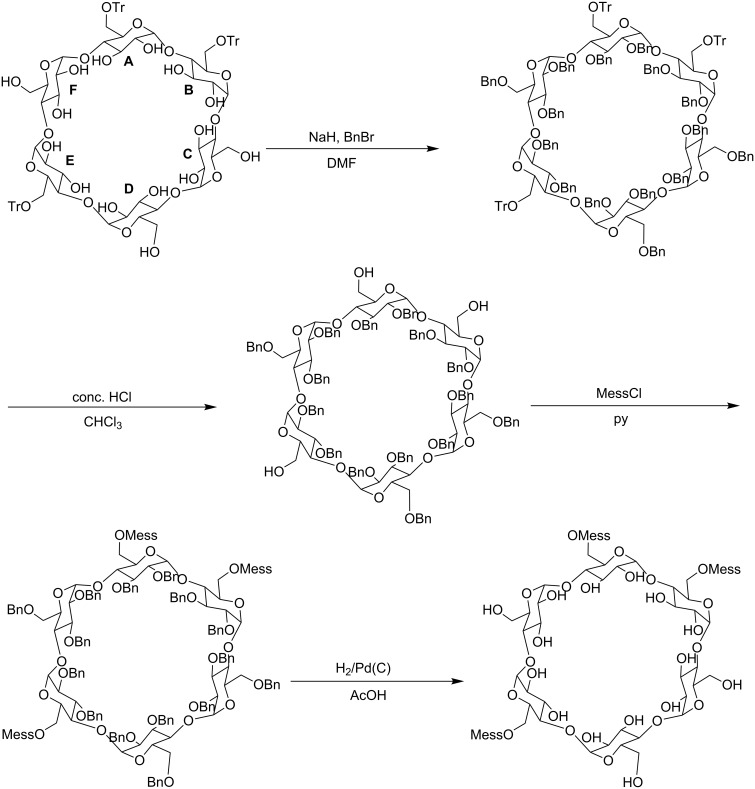
A pathway of the conversion of a regioisomer of tri-6-*O*-trityl-α-CD (ABE isomer) to the corresponding tri-6-*O*-mesitylenesulfonyl-α-CD. Letters A to F represent the glucopyranose units, numbered in a clockwise direction when viewed from the secondary hydroxy group side. Abbreviations: Tr: trityl, Bn: benzyl, and Mess: mesitylenesulfonyl.

**Figure 4:**
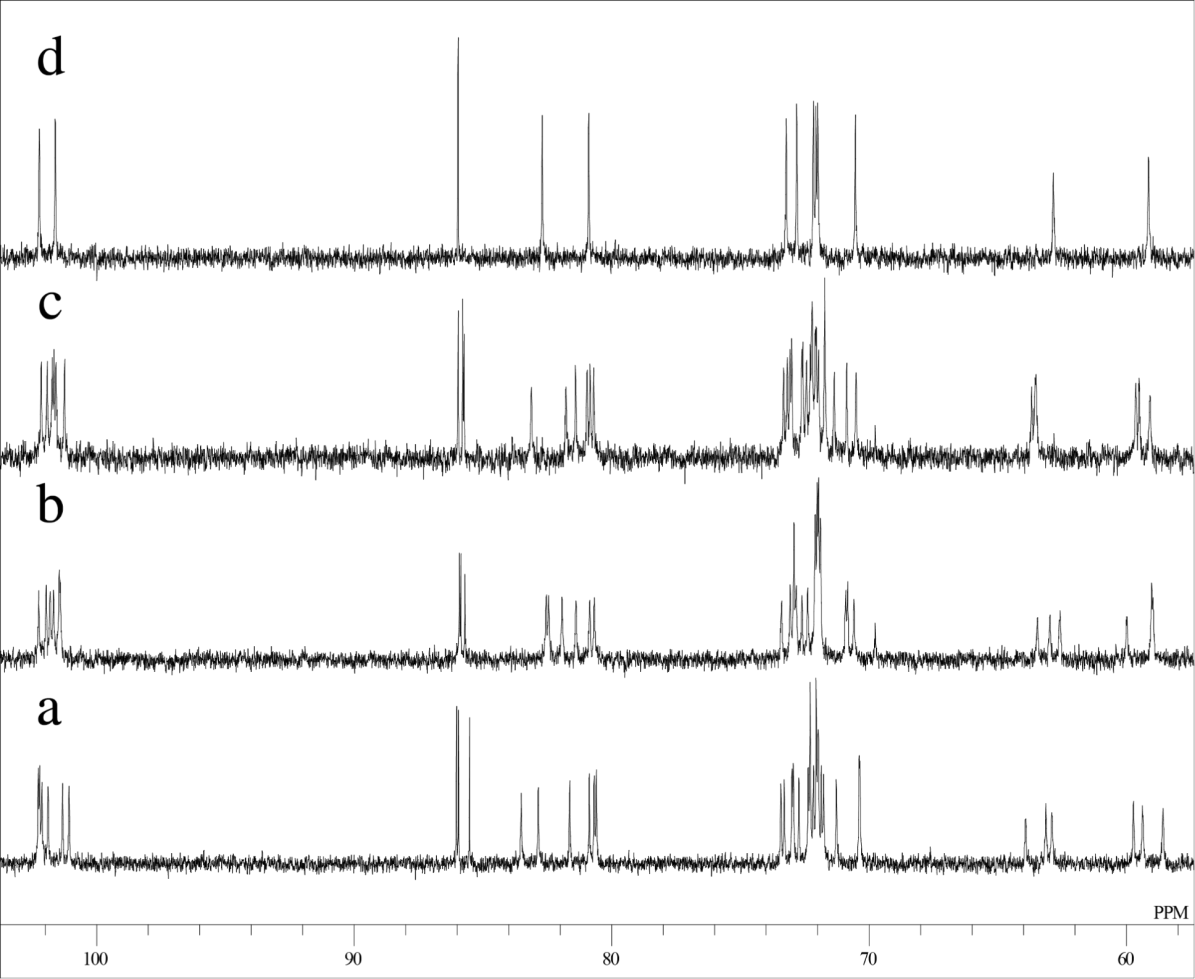
A part of ^13^C NMR spectra of tri-6-*O*-trityl-α-CD in DMSO-*d*_6_ at 50 °C. a) ABE-isomer, b) ABD-isomer, c) ABC-isomer, and d) ACE-isomer.

Mono-, di-, and tri-6-*O*-tritylates of α-CD were prepared by a reaction of α-CD with TrCl in pyridine at 55 °C according to the direction of literature [9], and separated by means of reversed-phase column chromatography. Three regioisomers of di-6-*O*-trityl-α-CD obtained gave well-separated peaks in UFLC (ultra-fast liquid chromatography) experiment (Figure 1), details of which will be described in the Experimental part. Four regioisomers of tri-6-*O*-trityl-α-CD also gave well-separated peaks in the UFLC chromatography (Figure 2), when acetonitrile/methanol/water (40/45/15, v/v/v) was used as an eluent. The di-6-*O*-trityl-α-CD regioisomers which gave the first, second, and third peaks in the UFLC chromatogram afforded well-defined ^13^C NMR spectra (Figure 3a, b, and c, respectively) in dimethyl sulfoxide-*d*_6_ (DMSO-*d*_6_) at 50 °C. Tanimoto, et al. [13], have already reported the ^13^C NMR spectra of the isomers measured in C_5_D_5_N. On the basis of a comparison of their spectra with ours, we assigned that the isomers which gave the first, second, and third peaks in the UFLC chromatogram are the AD-, AC-, and AB-isomers, respectively. This assignment was confirmed by UFLC analysis on the products of reactions of these three regioisomers with TrCl to give tri-6-*O*-trityl-α-CD. As Scheme 1 shows, the reaction of AD-isomer with TrCl gives only two tri-tritylates of ABD- and ABE-isomers. On the other hand, the additional tritylation of AB- and AC-isomers gives three (ABC-, ABD-, and ABE-) and four (ABC-, ABD-, ABE-, and ACE-) isomers, respectively. In practice, the additional tritylation of a ditritylate assigned to be AD-isomer by ^13^C NMR spectroscopy gave only two (the first and second) peaks in UFLC analysis, whereas those assigned to be AB- and AC-isomers by ^13^C NMR gave three (the first, second, and third) and four (the first, second, third, and fourth) peaks, respectively, in UFLC analysis. These results are in accord with the reaction pathway shown in Scheme 1. A similar method had been used for the identification of di- and tri-6-*O*-mesitylenesulfonyl-α-CD [20]. Scheme 1 also shows that the ACE-isomer is formed only by the reaction of the AC-isomer with TrCl, and we assigned that the fourth peak in UFLC chromatogram is due to the ACE-isomer. On the other hand, the ABC-isomer is not formed by the reaction of the AD-isomer with TrCl and is formed by the reactions of AC- and AB-isomers with TrCl. Thus, we assigned that the third peak is due to the ABC-isomer [20]. It is difficult to determine by UFLC analysis which of the ABD- or ABE-isomer gives the first or the second peak in UFLC. Here, we noted that 6^A^,6^B^,6^D^- and 6^A^,6^B^,6^E^-tri-6-*O*-mesitylenesulfonyl-α-CD’s have already been identified by ^1^H NMR spectroscopy [21–22]. Then, we tried to convert a regioisomer of tri-6-*O*-tritylates, which gave the first peak in UFLC, to tri-6-*O*-mesitylenesulfonyl-α-CD. The conversion pathway is shown in Scheme 2, where all the hydroxy groups of the regioisomer were substituted by benzyloxy groups, and the resulting per-benzylated compound was treated with conc. HCl to remove the trityl groups. The three hydroxy groups formed were mesitylenesulfonylated, and finally, the benzyl groups were removed by hydrogenation with Pd/C to form tri-6-*O*-mesitylenesulfonyl-α-CD. The retention time in UFLC and the ^1^H NMR spectrum of the obtained mesitylenesulfonylate coincided with those of authentic 6^A^,6^B^,6^E^-tri-6-*O*-mesitylenesulfonyl-α-CD, respectively. Similar conversion of tri-6-*O*-trityl-α-CD to tri-6-*O*-mesitylenesulfonyl-α-CD was carried out for a regioisomer which gave the second peak in UFLC, and the ^1^H NMR spectrum of the obtained mesitylenesulfonylate coincided with that of authentic 6^A^,6^B^,6^D^-tri-6-*O*-mesitylenesulfonyl-α-CD. Thus, we concluded that the regioisomers which gave the first and second peaks in UFLC are the ABE- and ABD-isomers, respectively. The ^13^C NMR spectra for the four regioisomers of tri-6-*O*-trityl-α-CD in DMSO-*d*_6_ at 50 °C are illustrated in Figure 4.

### Regioselectivity in the formation of di- and tri-6-*O*-trityl-α-CD

**Figure 5:**
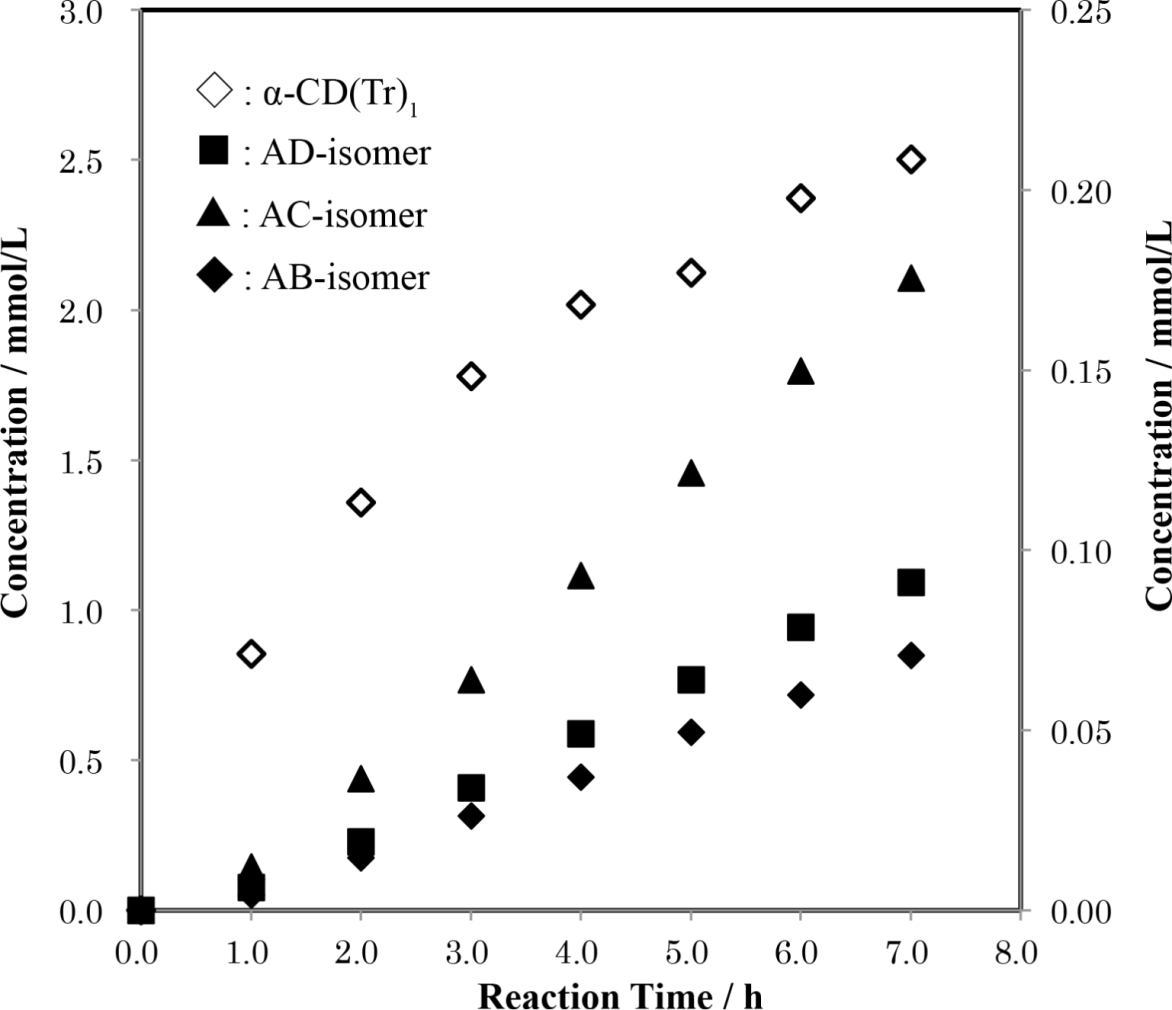
Time-course of the formation of mono- (left ordinate) and di-6-*O*-tritylates (right ordinate) of α-CD in a reaction of α-CD with TrCl in pyridine at 55 °C. Average mole ratio of AB:AC:AD = 0.40 ± 0.01:1.00:0.52 ± 0.02.

Dried α-CD was allowed to react with TrCl in pyridine at 55 °C, and aliquots were withdrawn at hourly intervals to determine the concentrations of products by means of the UFLC analysis. The concentrations of mono-6-*O*-trityl-α-CD, α-CD(Tr)_1_, and three regioisomers of ditritylates formed are plotted against reaction time in Figure 5. The concentration of α-CD(Tr)_1_ smoothly increased with time. On the other hand, the concentrations of di-6-*O*-tritylates of α-CD were low at the beginning of the reaction and gradually increased with time, suggesting that the ditritylates are formed by additional tritylation of the previously formed α-CD(Tr)_1_. If the reactivities of the C(6)-OH groups on the Glu residues of α-CD(Tr)_1_ are equal to each other, AB- and AC-isomers would be produced in equal amount and in twice that of AD-isomer. In practice, the molar ratio of the products was about 0.40 ± 0.01:1.00:0.52 ± 0.01 (AB:AC:AD) in every aliquot. The AB-isomer was produced considerably less than the AC-isomer.

**Figure 6:**
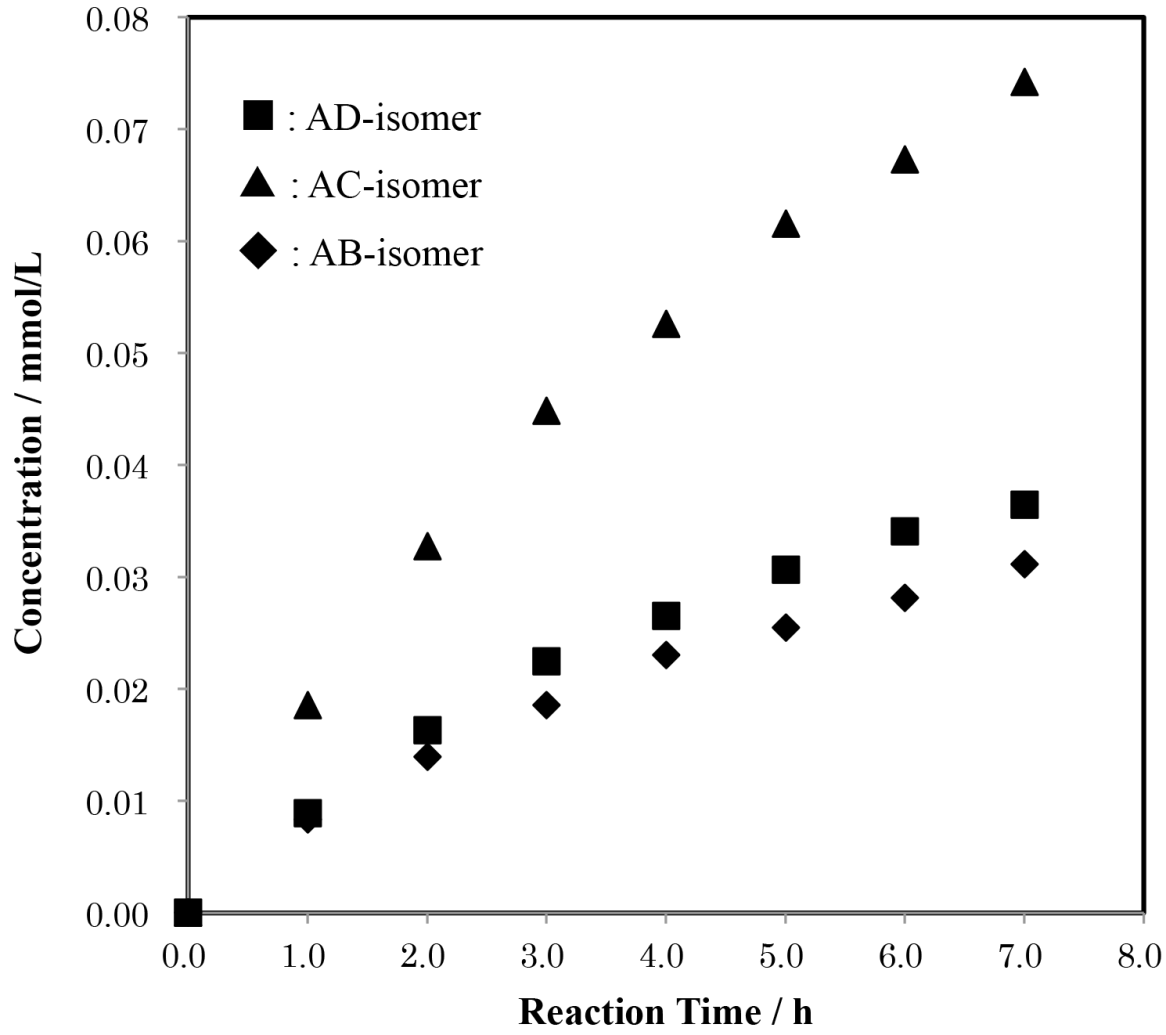
Time-course of the formation of the regioisomers of di-6-*O*-trityl-α-CD in a reaction of α-CD(Tr)_1_ with TrCl in pyridine at 55 °C. Average mole ratios of AB:AC:AD = 0.43 ± 0.01:1.00:0.50 ± 0.01.

In order to investigate the formation of the ditritylates in detail, α-CD(Tr)_1_ was directly tritylated in pyridine at 55 °C. Changes in concentration of three regioisomers of the ditritylates with time are illustrated in Figure 6. The average mole ratio of AB:AC:AD was 0.43 ± 0.01:1.00:0.50 ± 0.01, virtually the same as in the case when α-CD was used as reactant. We also tried to determine the concentrations of tri-6-*O*-tritylates of α-CD. However, the concentrations were so low that further tritylation of the ditritylates to tritritylates did not affect the mole ratio of the ditritylates. The small mole ratio of AB-isomer to AC-isomer suggests that the bulky trityl group on α-CD(Tr)_1_ retards the additional tritylation of the C(6)-OH on the adjacent Glu-B and/or Glu-F. Glu-B is adjacent to Glu-A in a clockwise direction, and Glu-F, in a counter-clockwise direction.

**Figure 7:**
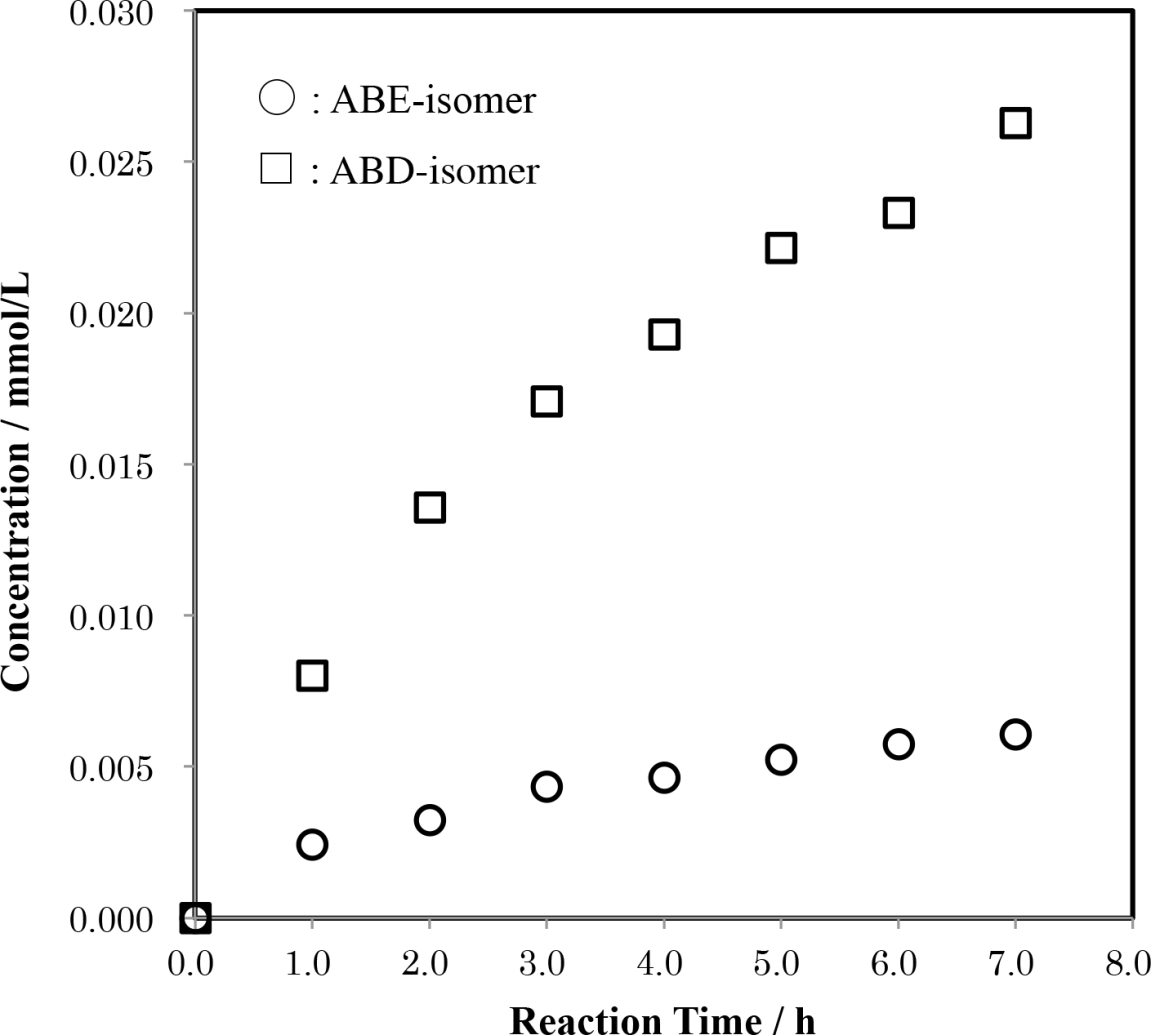
Time-course of the formation of the regioisomers of tri-6-*O*-trityl-α-CD in a reaction of AD-isomer with TrCl in pyridine at 55 °C. Average mole ratio of ABD**:**ABE = 1.00:0.25 ± 0.02.

Then, we carried out experiments using the AD-isomer as the reactant to elucidate which of the C(6)-OH of Glu-B or Glu-F is more restricted in the additional tritylation of α-CD(Tr)_1_. If the trityl groups of Glu-A and -D of the AD-isomer retard the additional tritylation of the adjacent C(6)-OH in a clockwise direction (Glu-B and -E, respectively), the ABD-isomer will be the minor product and the ABE-isomer the major one. To the contrary, if they retard the additional tritylation of the adjacent C(6)-OH in a counter-clockwise direction (Glu-F and -C, respectively), the ABE-isomer will be the minor product and the ABD-isomer the major one. The quantitative analysis of the product in this reaction revealed that the ABE-isomer is formed in a smaller amount than the ABD-isomer, with a mole ratio of 0.25 ± 0.02:1.00 (Figure 7). This result indicates that the trityl groups of Glu-A and Glu-D in the AD-isomer mainly retard the additional tritylation of the C(6)-OH of the adjacent Glu in a counter-clockwise direction (Glu-F and Glu-C, respectively). Thus, the trityl group of Glu-A in α-CD(Tr)_1_ will also mainly retard the additional tritylation of the C(6)-OH of the adjacent Glu in a counter-clockwise direction (Glu-F).

**Figure 8:**
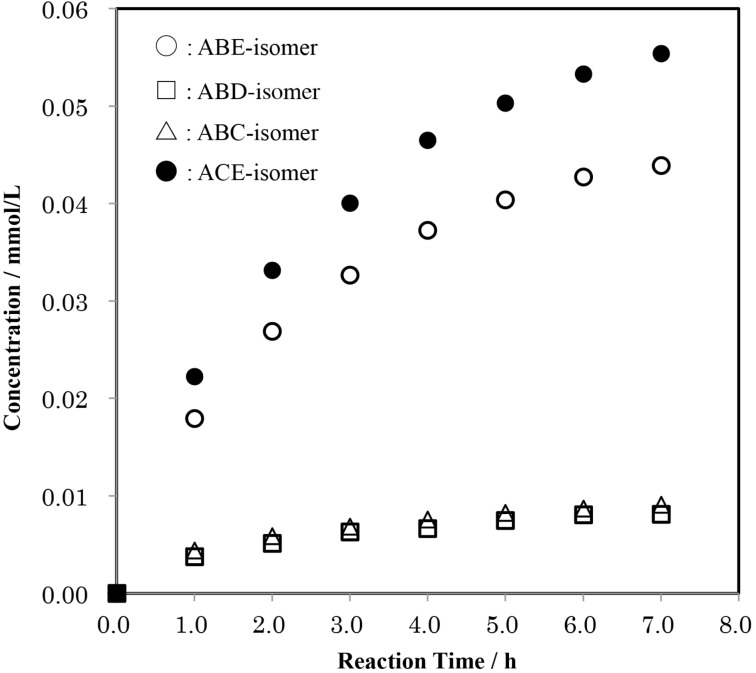
Time-course of the formation of the regioisomers of tri-6-*O*-trityl-α-CD in a reaction of AC-isomer with TrCl in pyridine at 55 °C. Average mole ratio of ABC:ABD:ABE:ACE = 0.17 ± 0.01:0.15 ± 0.01:0.80 ± 0.01:1.00.

Figure 8 shows the time-course of the formation of tri-6-*O*-tritylates of α-CD in the reaction of AC-isomer with TrCl in pyridine. In AC-isomer, Glu-B, -D, -E, and -F are chemically unequivalent to one another, and additional tritylation gives the four regioisomers of ABC-, ACD-, ACE-, and ACF. In this context, ACD- and ACF-isomers are identical to ABE- and ABD-isomers, respectively. If the additional tritylation occurs without regioselectivity, these regioisomers should be produced in an equal yield. In practice, the mole ratios of ABC-, ABD-, ABF-, and ACE-isomers in the reaction mixtures were virtually constant during the reaction time and equal to 0.17 ± 0.01:0.15 ± 0.01:0.80 ± 0.01:1.00. The additional tritylations of the C(6)-OHs on Glu-B and Glu-F, which are adjacent to Glu-C and Glu-A in a counter-clockwise direction, respectively, were again strongly retarded. Interestingly, the symmetrically substituted ACE-isomer was the major product, in clear contrast to the case of mesitylenesulfonylation of 6^A^,6^C^-di-*O*-mesitylenesulfonyl-α-CD, where the ACE-isomer is the minor product [23].

**Figure 9:**
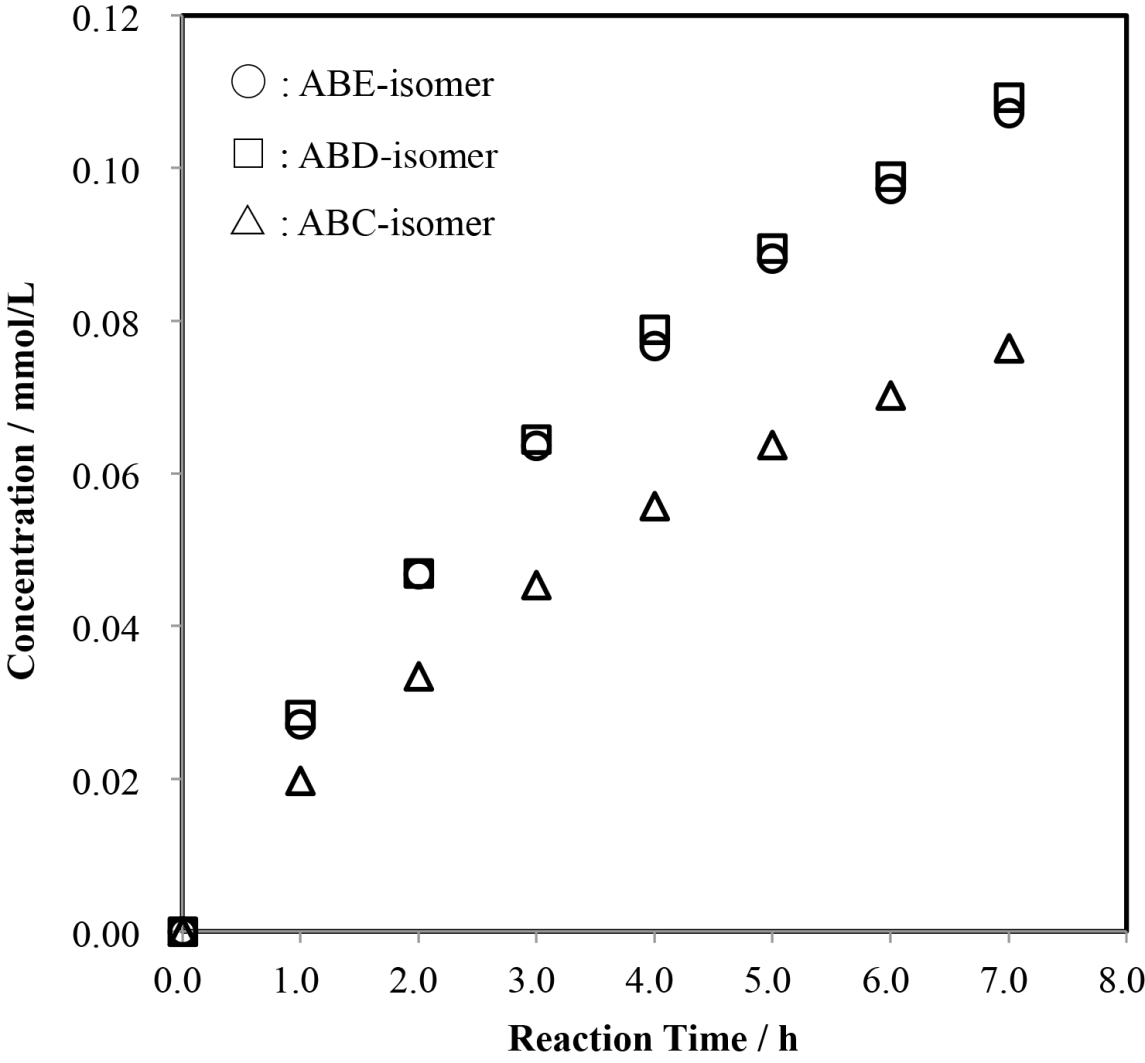
Time-course of the formation of the regioisomers of tri-6-*O*-trityl-α-CD in a reaction of the AB-isomer with TrCl in pyridine at 55 °C. Average mole ratios of ABC:ABD:ABE = 0.71 ± 0.01:1.00:0.98 ± 0.01.

Figure 9 shows the time-course of the formation of tri-6-*O*-tritylates of α-CD in the reaction of the AB-isomer with TrCl in pyridine. In the AB-isomer, the additional tritylation of Glu-C and -F gives the same product, the ABC-isomer. Thus, if the tritylation occurs without regioselectivity, the mole ratio of ABC-, ABD-, and ABE-isomers would be 2:1:1. In practice, the mole ratio was 0.71 ± 0.01:1.00:0.98 ± 0.01. Again, the tritylation of Glu-C and/or Glu-F, which are adjacent to Glu-B and Glu-A, respectively, was strongly retarded.

### ^1^H NMR spectroscopy of the AD-isomer

**Figure 10:**
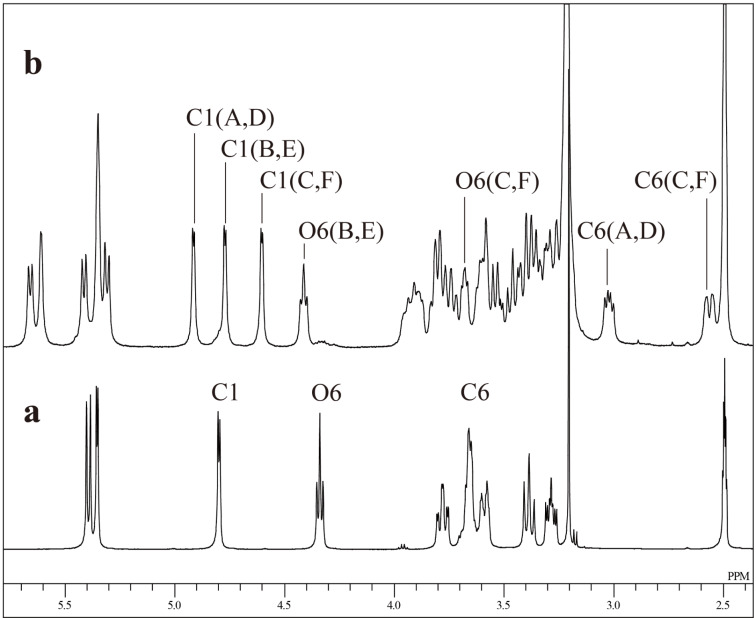
^1^H NMR spectra of α-CD (a) and the A,D-isomer (b) in DMSO-*d*_6_ at 50 °C.

**Figure 11:**
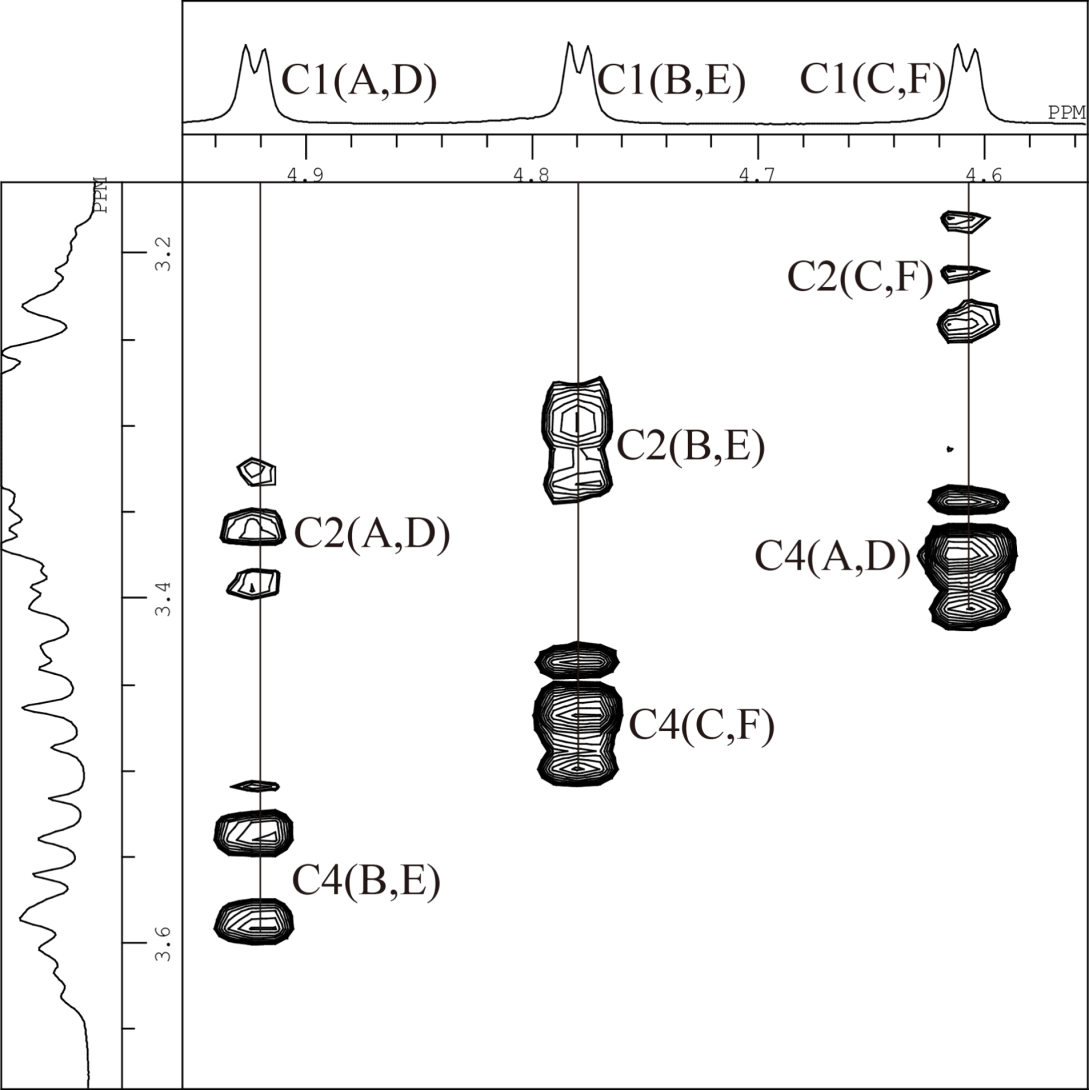
A part of 2D ROESY spectrum of the A,D-isomer in DMSO-*d*_6_ at 50 °C.

All the above results shows that the trityl group introduced to the C(6)-O of α-CD retards additional tritylation of the C(6)-OH of Glu adjacent to the previously tritylated Glu in a counter-clockwise direction. In order to confirm this suggestion, we estimated the orientation of the trityl groups in the AD-isomer by means of ^1^H NMR spectroscopy in DMSO-*d*_6_ (Figure 10). The AD-isomer is symmetrical and it is relatively easy to assign the ^1^H NMR signals. The spectrum of the AD-isomer was fully assigned by means of 2D COSY, 2D ROESY, and 2D TOCSY spectra (Table 1). A 2D ROESY measurement gave a key spectrum (Figure 11) used for the assignment. The signal of the C(1)-H for Glu-A and -D in the F2 axis gave cross-peaks not only with the C(2)-H (δ 3.36) of the same Glu, but also with the C(4)-H (δ 3.56) of the adjacent Glu-B and -E. In the same manner, the signal of C(1)-H for Glu-B and -E gave a cross-peak with the C(4)-H (δ 3.47) of the adjacent Glu-C and -F, and that for Glu-C and -F gave a cross-peak with the C(4)-H (δ 3.37) of the adjacent Glu-D and -A. It is worth noting that the signal (δ 3.72) of the O(6)-H for Glu-C and -F was fairly up-field shifted (Δδ = −0.62 ppm) compared with that (δ 4.34) of α-CD. On the other hand, the signal of the O(6)-H for Glu-B and -E (δ 4.45) was slightly down-field shifted (Δδ = 0.11 ppm). The signal (δ 2.55) of one of the C(6)-H’s of Glu-C and -F was also significantly shifted up-field (Δδ = −1.11 ppm) compared with that (δ 3.66) of α-CD. These significant up-field shifts of the signals of the O(6)-H and C(6)-H for Glu-C and -F are attributable to the ring current of the phenyl moieties of the trityl groups on Glu-A and -D. Glu-C and -F are located at counter-clockwise directions from Glu-D and -A, respectively. Thus, it is reasonable to conclude that the trityl groups on Glu-A and -D are oriented to Glu-F and -C, respectively, and sterically retard additional tritylation of the C(6)-OHs on Glu-F and -C. Similar steric hindrance will occur in the tritylation of α-CD(Tr)_1_ as well as in the tritylations of AB- and AC-regioisomers.

**Table 1:** Chemical shift of A,D-α-CD(tr)_2_ in DMSO-*d*_6_ at 50 °C.

	Chemical shift / ppm		

	Glu(A,D)	Glu(B,E)	Glu(C,F)

C(1)-H	4.92	4.78	4.61
C(2)-H	3.36	3.31	3.23
C(3)-H	3.80	3.82	3.72
C(4)-H	3.37	3.56	3.47
C(5)-H	3.89	3.75	3.68
C(6)-H(a)	3.40	3.96	3.30
C(6)-H(b)	3.02	3.62	2.55
O(2)-H	5.33	5.44	5.68
O(3)-H	5.63	5.37	5.37
O(6)-H	–	4.45	3.72
Tr(*o*)-H	7.32	–	–
Tr(*m*)-H	7.21	–	–
Tr(*p*)-H	7.13	–	–

In conclusion, the bulky trityl group on a tritylate of α-CD is oriented to the C(6)-OH on the adjacent glucopyranose in a counter-clockwise direction and sterically retards the additional tritylation of the C(6)-OH.

## Experimental

### Materials

The α-CD was supplied by Ensuiko Sugar Refining Co., Ltd., and dried overnight in vacuo at 110 °C before use. TrCl (Tokyo Chemical Industry Co., Ltd.), benzyl bromide and mesitylenesulfonyl chloride (Wako Pure Chemical Industries Ltd.) were used without further purification. Commercially available NaH in oil (50–77%) and Pd/C (Pd 10%) were used for the conversion of tri-6-*O*-trityl-α-CD to tri-6-*O*-mesitylenesulfonyl-α-CD. Reagent-grade pyridine was dried over CaH_2_ and distilled in the presence of CaH_2_ before use. *N*,*N*-Dimethylformamide (DMF) was also dried over CaH_2_ and distilled before use. Acetonitrile for high performance liquid chromatography was purchased from Wako Pure Chemical Industries Ltd. DMSO-*d*_6_ containing 0.05% v/v tetramethylsilane (Cambridge Isotope Laboratiories, Inc., 99.9 atom % D) was used for ^1^H NMR measurements.

### Apparatus

The tritylates of α-CD in a reaction mixture were quantitatively determined by means of a Shimadzu prominence UFLC system, which was composed of a pair of pumps (LC-20 AD), an auto-sampler (SIL-20 AC_HT_), a column oven (CTO-20AC), a photodiode array detector (SPD-M20A), and a system controller (CBM-20A). Shim-pack XR-ODS (100 mm × 3.0 mm i.d. was used as a column, through which acetonitrile/water or acetonitrile/methanol/water was passed as an eluent. The flow rate of eluent was 0.5 mL/min, and the eluate was detected at 260 nm. The column temperature was maintained at 40 °C. Linear standard curves were prepared between the peak area of the tritylates and their concentrations. The ^1^H and ^13^C NMR spectra of the tritylates in DMSO-*d*_6_ were recorded on a JEOL Model JNM-A400 FT NMR spectrometer (400 MHz for ^1^H and 100 MHz for ^13^C) with a sample tube of 5.0 mm diameter at 55 °C. Tetramethylsilane was used as an internal reference. High resolution ESIMS data were obtained using a Waters Synapt G2 mass spectrometer.

### Synthesis of 6-*O*-tritylates of α-CD

6-*O*-Tritylates of α-CD were prepared by a reaction of α-CD with TrCl in pyridine at 55 °C according to a literature procedure [9], and separated by means of column chromatography with a Lober column LiChroprep RP-18 (Merck, 25 × 310 mm). Mono-, di-, and tri-6-*O*-tritylates were separated by the use of 30%, 40–50%, and 60% aqueous acetonitrile solutions, respectively, as eluents. Thin-layer chromagography (TLC) was carried out with Merck silica gel 60 F_254_.

**Mono-6-*O*-trityl-α-CD**: ^13^C NMR (100 MHz, DMSO-*d*_6_) δ 144.03, 128.30, 127.57, 126.72, 102.28, 101.89, 101.85, 101.75, 85.97, 82.70, 82.03, 81.89, 81.84, 80.94, 73.21–73.12, 72.80, 72.21, 72.09–72.05, 71.89, 70.41, 63.08, 60.13, 59.96, 59.84, 58.96 ppm; HRMS–ESI *m/z*: [M + Na]^+^ calcd for C_55_H_74_O_30_Na^+^, 1237.4163; found, 1237.4150.

### Conversion of tri-6-O-trityl-α-CD to tri-6-O-mesitylenesuflonyl-α-CD

A regioisomer of tri-6-*O*-trityl-α-CD (605 mg, 0.356 mmol) which gave the first peak in UFLC was dissolved in DMF (30 mL). NaH in oil (1.00 g) and benzyl bromide (3.10 g, 18.1 mmol) were added to the solution in an ice bath, and the solution was stirred overnight. TLC on silica gel (hexane/ethyl acetate, 2:1) showed a product, having an *R*_f_ value of 0.60. The reaction mixture was diluted with CHCl_3_ (100 mL) and washed with water (100 mL, 4 times). The CHCl_3_ layer was dried over CaSO_4_ and evaporated to dryness. The residue was chromatographed over Merck silica gel 60, using hexane/ethyl acetate (2:1) as an eluent. The crude tri-6-*O*-trityl-per-*O*-benzyl-α-CD (1.32 g) obtained was dissolved in CHCl_3_ (100 mL), and the solution was vigorously shaken with concentrated HCl (30 mL) for 10 min to remove the trityl groups. TLC on silica gel (hexane/ethyl acetate, 1:1) showed a product, having an *R*_f_ value of 0.55. The CHCl_3_ layer was washed with water (50 mL, 3 times), dried over CaSO_4_, and evaporated to dryness. The residue was chromatographed over silica gel, using hexane/ethyl acetate (1:1) as an eluent to give a fairly pure product of trihydroxy-per-*O*-benzyl-α-CD (0.48 g, 0.21 mmol). The product was reacted with mesitylenesulfonyl chloride (0.62 g, 2.84 mmol) in pyridine (20 mL). TLC on silica gel (hexane/ethyl acetate 1:1) showed a product, having an *R*_f_ value of 0.91. The reaction mixture was evaporated to dryness, and the residue was dissolved in 50 mL of hexane/ethyl acetate (2:1). The solution was washed with water (50 mL, 3 times). The organic layer was dried over CaSO_4_ and evaporated to dryness. The residue was chromatographed over silica gel, using hexane/ethyl acetate (2:1) as an eluent to give a fairly pure tri-6-*O*-mesitylenesulfonyl-per-*O*-benzyl-α-CD (0.51 g). The product was dissolved in 5 mL of acetic acid and hydrogenated in the presence of Pd/C (0.21 g) to remove the benzyl groups of the product. TLC on silica gel (butanone/ethanol/H_2_O, 7:1:1) showed a product, having an *R*_f_ value of 0.47, which was equal to that of authentic tri-6-*O*-mesitylenesulfonyl-α-CD. The reaction mixture was filtered, and the filtrate was evaporated to dryness to give a fairly pure tri-6-*O*-mesitylenesulfonyl-α-CD (0.26 g, 0.17 mmol). The retention time of the product in UFLC with 43% aqueous acetonitrile as an eluent coincided with that of authentic A,B,E-tri-6-*O*-mesitylenesulfonyl-α-CD [23]. The ^1^H NMR spectrum of the product in DMSO-*d*_6_ at 50 °C also coincided with that of authentic A,B,E-tri-6-*O*-mesitylenesulfonyl-α-CD [23]. Thus, it was concluded that a regioisomer of tri-6-*O*-trityl-α-CD which gave the first peak in UFLC is A,B,E-tri-6-*O*-trityl-α-CD. Similar conversion of a regioisomer of tri-6-*O*-trityl-α-CD, which gave the second peak in UFLC, to tri-6-*O*-mesitylenesulfonyl-α-CD was carried out, and the resulting product was confirmed to be A,B,D-tri-6-*O*-mesitylenesulfonyl-α-CD by ^1^H NMR spectroscopy.

### Reaction of α-CD or α-CD(Tr)_1_ with TrCl and product analysis

α-CD (202 mg, 0.208 mmol) or α-CD(Tr)_1_ (102 mg, 0.084 mmol) was dissolved in pyridine (60 mL). The solution was boiled to remove trace amounts of water as an azeotropic mixture, and the resulting solution (50 mL) was stirred in an oil bath (55 °C). TrCl (111 mg, 0.399 mmol for α-CD or 41.5 mg, 0.149 mmol for α-CD(Tr)_1_) was added to the stirred solution. At hourly intervals, 2.0 mL aliquots were withdrawn and added to 0.5 mL water to stop the reaction. The resulting solution was evaporated to dryness, and the residue was dissolved in 2.0 mL methanol. Concentrations of products in the methanol solutions were determined by UFLC with acetonitrile/water (30:70 and 45:55, v/v, for the separation of mono- and ditritylates, respectively) as mobile phase. Retention times of α-CD(Tr)_1_ and the AD-, AC-, and AB-isomers were ca. 3.8, 2.7, 6.6, and 9.9 min, respectively.

**6^A^,6^B^-Di-*O*-trityl-α- CD (AB-isomer): **^13^C NMR (DMSO-*d*_6_) δ 144.26, 143.80, 128.19, 128.07, 127.66, 127.41, 126.71, 126.58, 102.07, 102.00, 101.97, 101.79, 101.59, 101.37, 86.03, 85.65, 83.27, 81.67, 81.59, 81.38, 81.34, 80.76, 73.32, 73.22, 73.14, 72.83, 72.36, 72.30, 72.24, 72.17, 72.11, 72.02, 71.78, 71.22, 70.37, 63.78, 63.19, 59.84, 59.58, 59.17 ppm; HRMS–ESI (*m*/*z*): [M + Na]^+^ calcd for C_74_H_88_O_30_Na^+^, 1479.5258; found, 1479.5217.

**6^A^,6^C^-Di-*O*-trityl-α- CD (AC-isomer): **^13^C NMR (DMSO-*d*_6_) δ 144.06, 143.99, 128.34, 128.27, 127.63, 127.53, 126.76, 126.67, 102.40, 102.10, 101.90, 101.76, 101.59, 86.03, 85.97, 83.12, 82.36, 81.79, 81.70, 81.00, 80.92, 73.25, 73.16, 72.94, 72.74, 72.23, 72.07, 70.56, 70.40, 63.28, 63.04, 59.98, 59.83, 59.06, 59.02 ppm;. HRMS–ESI (*m*/*z*): [M + Na]^+^ calcd for C_74_H_88_O_30_Na^+^, 1479.5258; found, 1479.5217.

**6^A^,6^D^-Di-*O*-trityl-α- CD (AD-isomer): **^13^C NMR (DMSO-*d*_6_) δ 143.89, 128.29, 127.51, 126.69, 102.41, 101.87, 101.45, 86.06, 83.09, 81.52, 80.82, 73.35, 73.02, 72.89, 72.32, 72.12, 72.00, 71.85, 70.36, 63.32, 60.02, 58.77 ppm; HRMS–ESI (*m*/*z*): [M + Na]^+^ calcd for C_74_H_88_O_30_Na^+^, 1479.5258; found, 1479.5217.

### Reaction of AD-, AC-, or AB-isomers with TrCl and product analysis

The AD-isomer (78 mg, 0.053 mmol), AC-isomer (150 mg, 0.103 mmol), or AB-isomer (151 mg, 0.104 mmol) was dissolved in pyridine (35 mL). The solution was boiled to remove a trace amount of water as an azeotropic mixture, and the resulting solution (25 mL) was stirred in an oil bath (55 °C). TrCl (23 mg, 0.083 mmol for AD-isomer, 53 mg, 0.189 mmol for AC-isomer, or 58 mg, 0.207 mmol for AB-isomer) was added to the stirred solution. At hourly intervals, 2.0 mL aliquots were withdrawn, added to 0.5 mL water to stop the reaction. The resulting solution was evaporated to dryness, and the residue was dissolved in 2.0 mL methanol. Concentrations of products in the methanol solutions were determined by UFLC with acetonitrile/methanol/water (40:45:15, v/v/v) as mobile phase for the separation of tri-tritylates formed. Retension times of ABE-, ABD-, ABC-, and ACE-isomers were ca. 6.2, 7.5, 9.4 and 11.3 min, respectively.

**6^A^,6^B^,6^C^-Tri-*O*-trityl-α-CD (ABC-isomer): **^13^C NMR (DMSO-*d*_6_) δ 144.32, 144.20, 143.66, 128.24, 128.13, 127.93, 127.71, 127.39, 126.80, 126.61, 126.40, 102.16, 101.93, 101.73, 101.66, 101.59, 101.25, 85.97, 85.78, 85.73, 83.12, 81.78, 81.40, 80.95, 80.83, 80.69, 73.32, 73.18, 73.07, 73.00, 72.62, 72.57, 72.42, 72.28, 72.20, 72.09, 72.05, 71.96, 71.72, 71.35, 70.87, 70.51, 63.69, 63.52, 59.65, 59.51, 59.08 ppm; HRMS–ESI (*m*/*z*): [M + Na]^+^ calcd for C_93_H_102_O_30_Na^+^, 1721.6345; found, 1721.6067.

**6^A^,6^B^,6^D^-Tri-*O*-trityl-α-CD (ABD-isomer): **^13^C NMR (DMSO-*d*_6_) δ 144.11, 143.79, 128.29, 128.15, 128.09, 127.54, 127.45, 127.38, 126.62, 102.25, 101.97, 101.82, 101.68, 101.46, 101.42, 85.92, 85.86, 85.69, 82.53, 82.44, 81.93, 81.38, 80.85, 80.67, 73.39, 73.07, 72.92, 72.83, 72.60, 72.38, 72.09, 72.01, 71.96, 71.89, 70.91, 70.83, 70.58, 69.76, 63.45, 62.97, 62.58, 59.99, 59.02, 58.97 ppm; HRMS–ESI) (*m*/*z*): [M + Na]^+^ calcd for C_93_H_102_O_30_Na^+^, 1721.6345; found, 1721.6067.

**6^A^,6^B^,6^E^-Tri-*O*-trityl-α-CD (ABE-isomer): **^13^C NMR (DMSO-*d*_6_) δ 144.25, 143.69, 143.56, 128.28, 128.15, 127.96, 127.63, 127.42, 127.31, 126.68, 126.62, 126.53, 102.27, 102.22, 102.13, 101.89, 101.33, 101.08, 86.02, 85.95, 85.52, 83.51, 82.85, 81.63, 80.86, 80.68, 80.59, 73.42, 73.30, 72.99, 72.94, 72.73, 72.36, 72.29, 72.16, 72.05, 71.97, 71.86, 71.77, 71.27, 70.38, 70.35, 63.91, 63.13, 62.90, 59.73, 59.38, 58.58 ppm; HRMS–ESI (*m*/*z*): [M + Na]^+^ calcd for C_93_H_102_O_30_Na^+^, 1721.6345; found, 1721.6067.

**6^A^,6^C^,6^E^-Tri-*O*-trityl-α-CD (ACE-isomer): **^13^C NMR (DMSO-*d*_6_) δ 143.98, 128.29, 127.54, 126.68, 102.23, 101.61, 85.97, 82.69, 80.89, 73.21, 72.81, 72.16, 72.06, 71.99, 70.53, 62.85, 59.14 ppm; HRMS–ESI (*m*/*z*): [M + Na]^+^ calcd for C_93_H_102_O_30_Na^+^, 1721.6345; found, 1721.6067.

## Supporting Information

File 1 ^1^H NMR spectra of mono-, di-, and tri-*O*-trityl-α-CD, together 2D COSY and TOCSY spectra of the AD-isomer.
