# Supplementary material for: Qualitative evaluation of regioselectivity in the formation of di- and tri-6-O-tritylates of α-cyclodextrin
Source: Beilstein J Org Chem. 2015 Sep 2;11:1530–40. doi: 10.3762/bjoc.11.168 (PMC4578407; doi:10.3762/bjoc.11.168)

Supporting Information
for

Qualitative evaluation of regioselectivity in the formation of di- and tri-6-*O*-tritylates of α-cyclodextrin

Keisuke Yoshikiyo*, Yoshihisa Matsui and Tatsuyuki Yamamoto

Address: Faculty of Life and Environmental Science, Shimane University, 1060 Nishikawatsu, Matsue, Shimane, 690-8504, Japan

Email: Keisuke Yoshikiyo* - yoshikiyo@life.shimane-u.ac.jp

*Corresponding author

Table of contents

^{1}H NMR spectrum of Mono-6-*O*-trityl-α-CD S2

^{1}H NMR spectrum of 6^{A},6^{B}-di-*O*-trityl-α- CD (AB-isomer) S3

^{1}H NMR spectrum of 6^{A},6^{C}-di-*O*-trityl-α- CD (AC-isomer) S4

^{1}H NMR spectrum of 6^{A},6^{D}-di-*O*-trityl-α- CD (AD-isomer) S5

^{1}H NMR spectrum of 6^{A},6^{B},6^{C}-tri-*O*-trityl-α-CD (ABC-isomer) S6

^{1}H NMR spectrum of 6^{A},6^{B},6^{D}-tri-*O*-trityl-α-CD (ABD-isomer) S7

^{1}H NMR spectrum of 6^{A},6^{B},6^{E}-tri-*O*-trityl-α-CD (ABE-isomer) S8

^{1}H NMR spectrum of 6^{A},6^{C},6^{E}-tri-*O*-trityl-α-CD (ACE-isomer) S9

2D COSY spectrum of the AD-isomer in DMSO-d_6 at 50 °C S10

2D TOCSYspectrum of the AD-isomer in DMSO-d_6 at 50 °C S11

Mono-6-*O*-trityl-α-CD

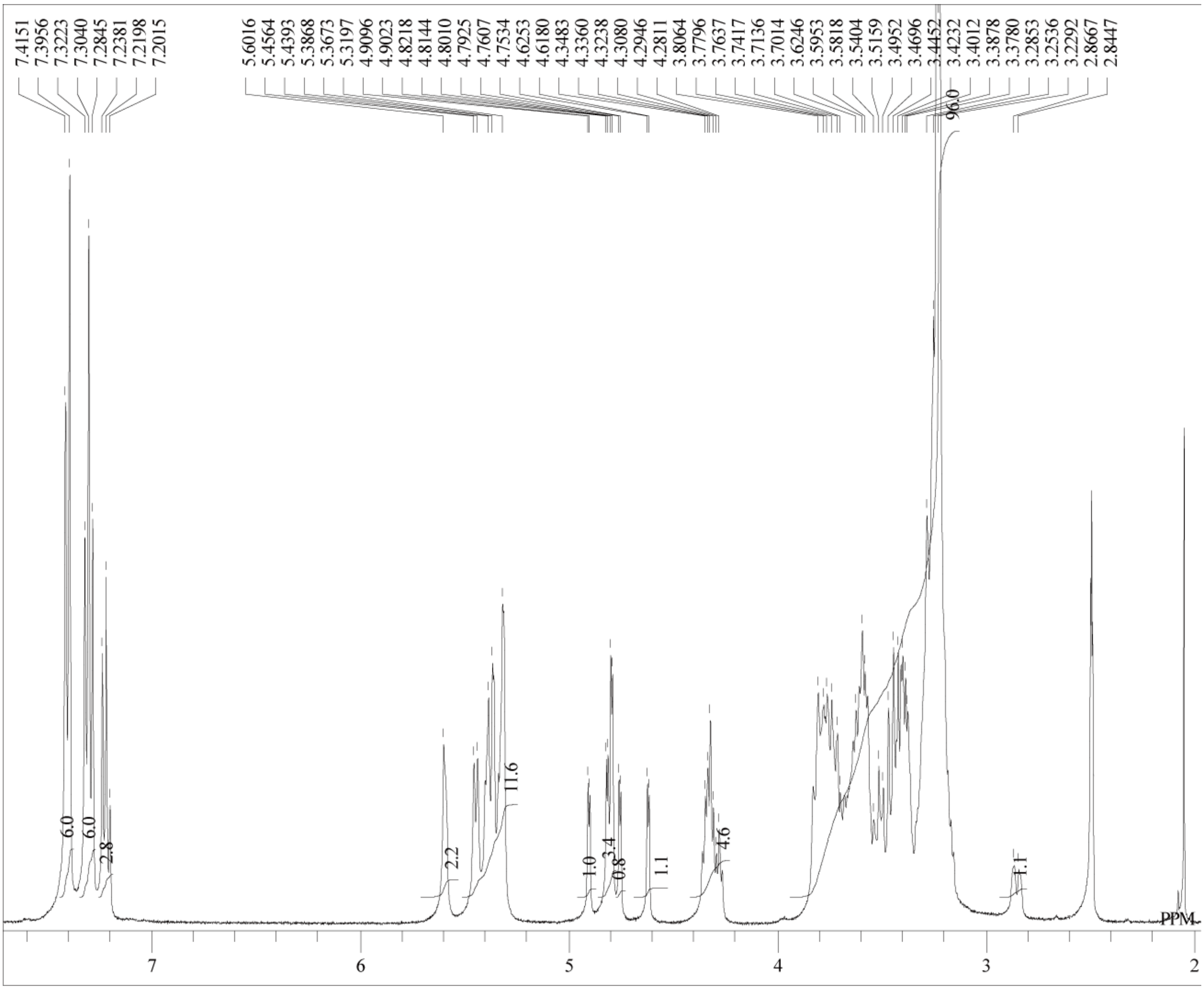

6[A],6[B]-Di-*O*-trityl-α- CD (AB-isomer)

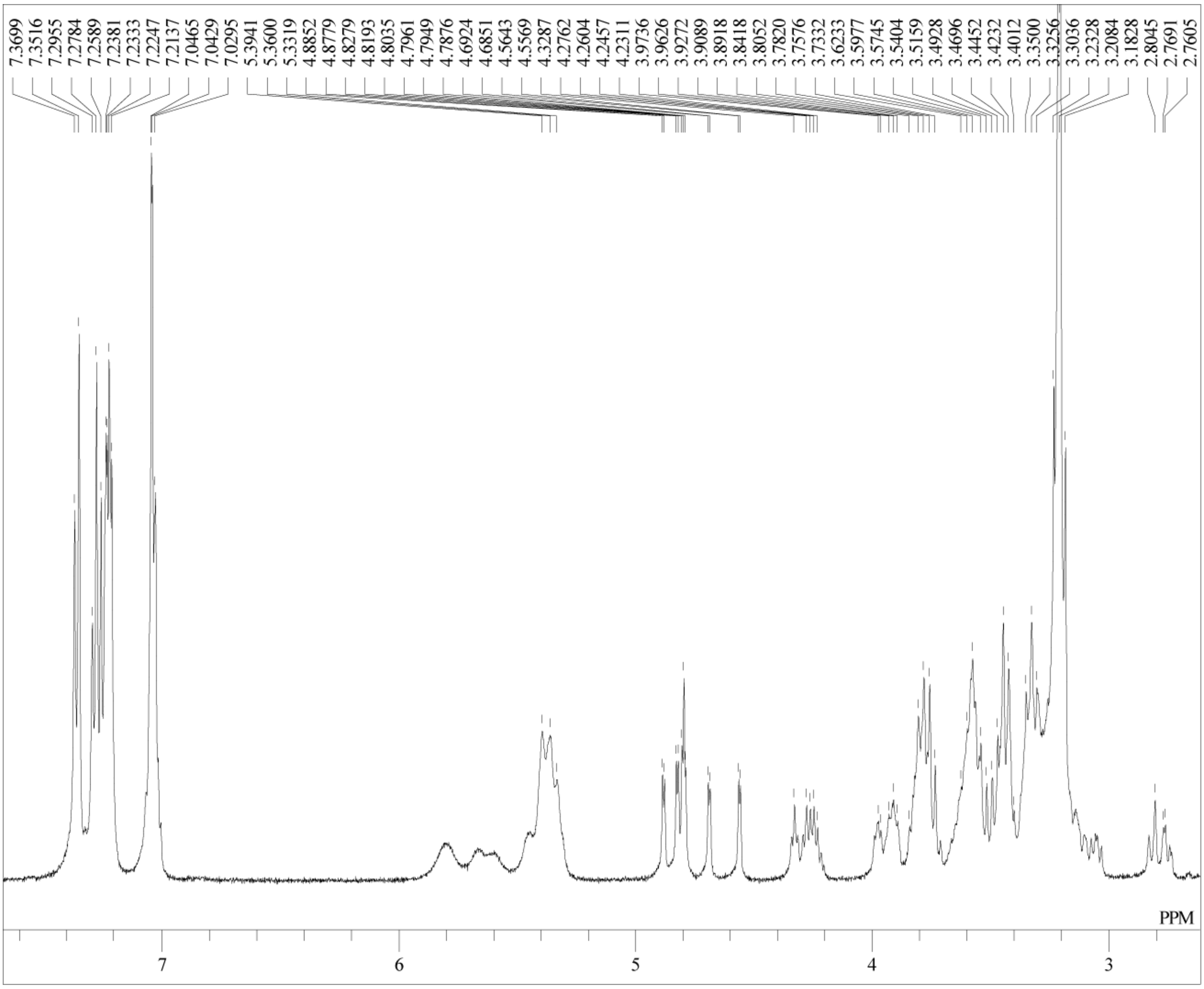

6^{A},6^{C}-Di-*O*-trityl-α- CD (AC-isomer)

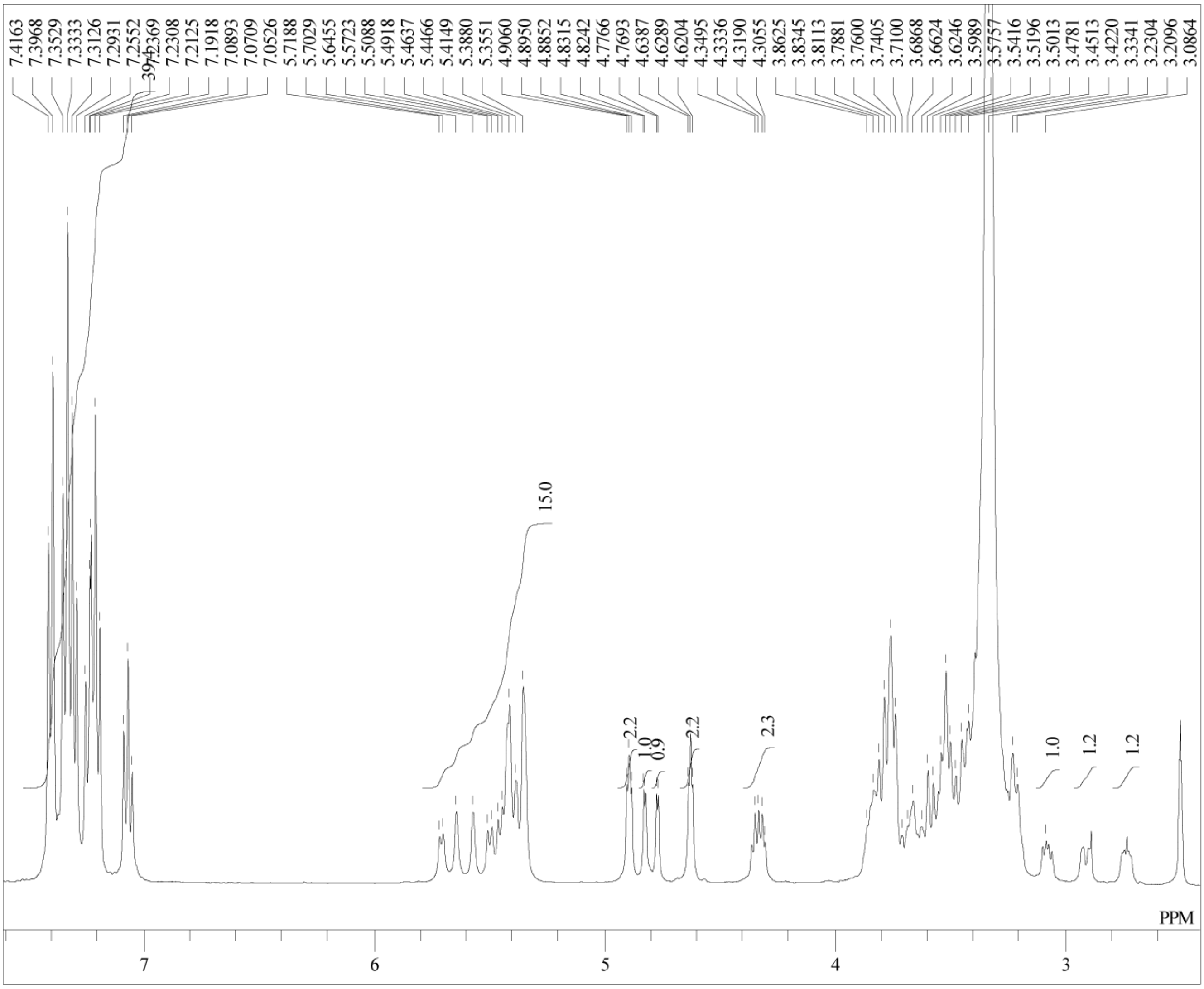

6^{A},6^{D}-Di-*O*-trityl-α- CD (AD-isomer)

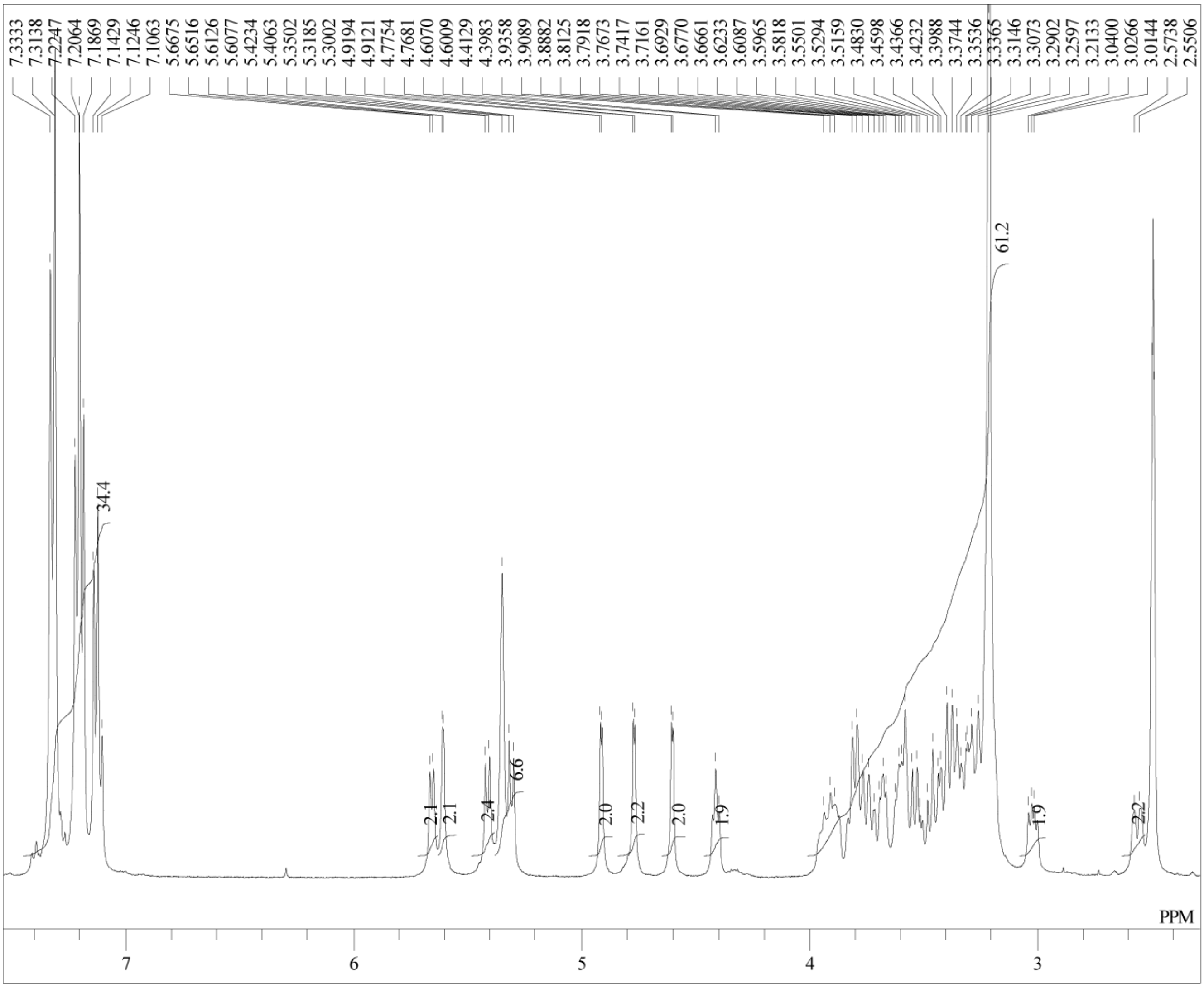

6^{A},6^{B},6^{C}-Tri-*O*-trityl-α-CD (ABC-isomer)

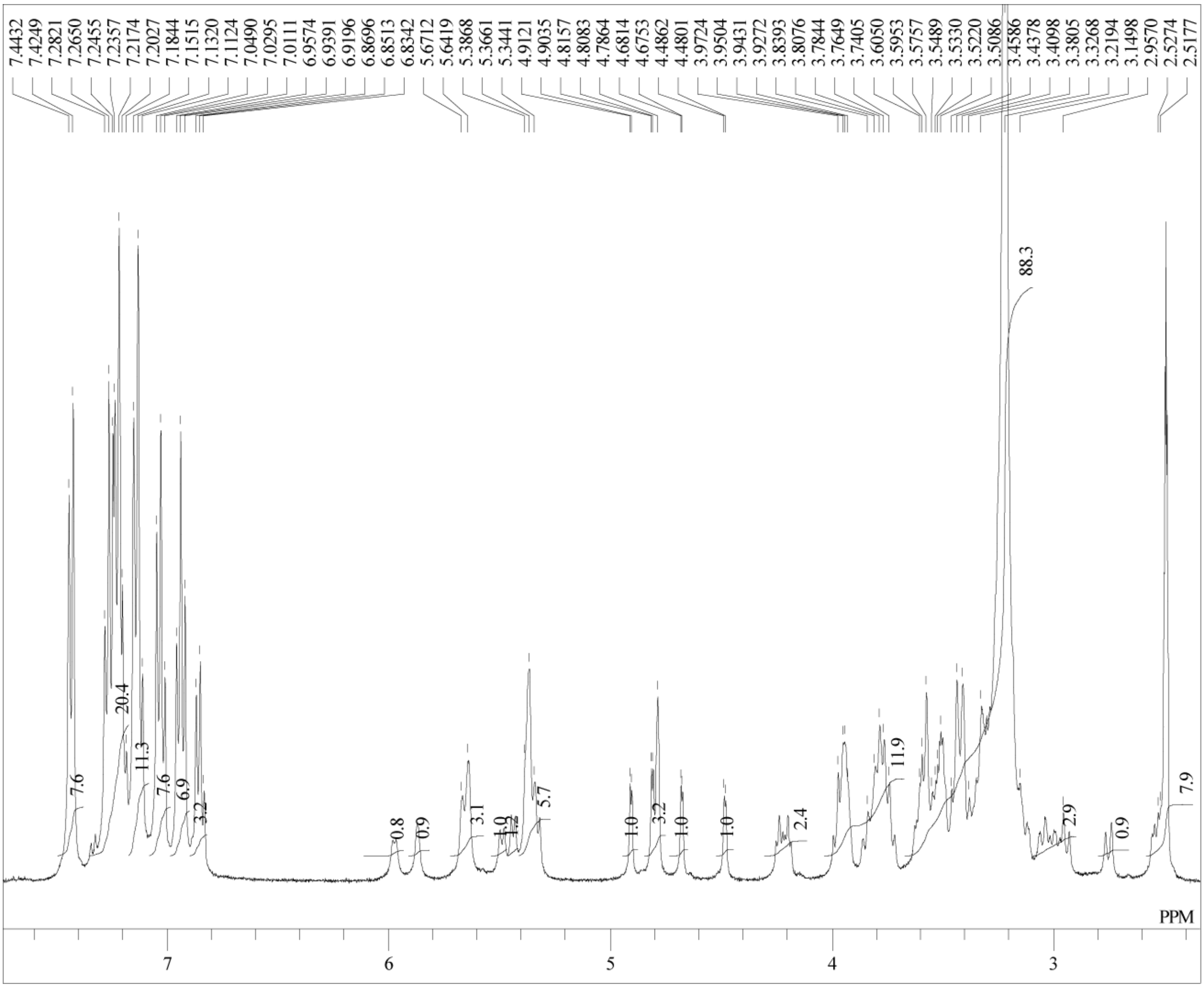

6^{A},6^{B},6^{D}-Tri-*O*-trityl-α-CD (ABD-isomer)

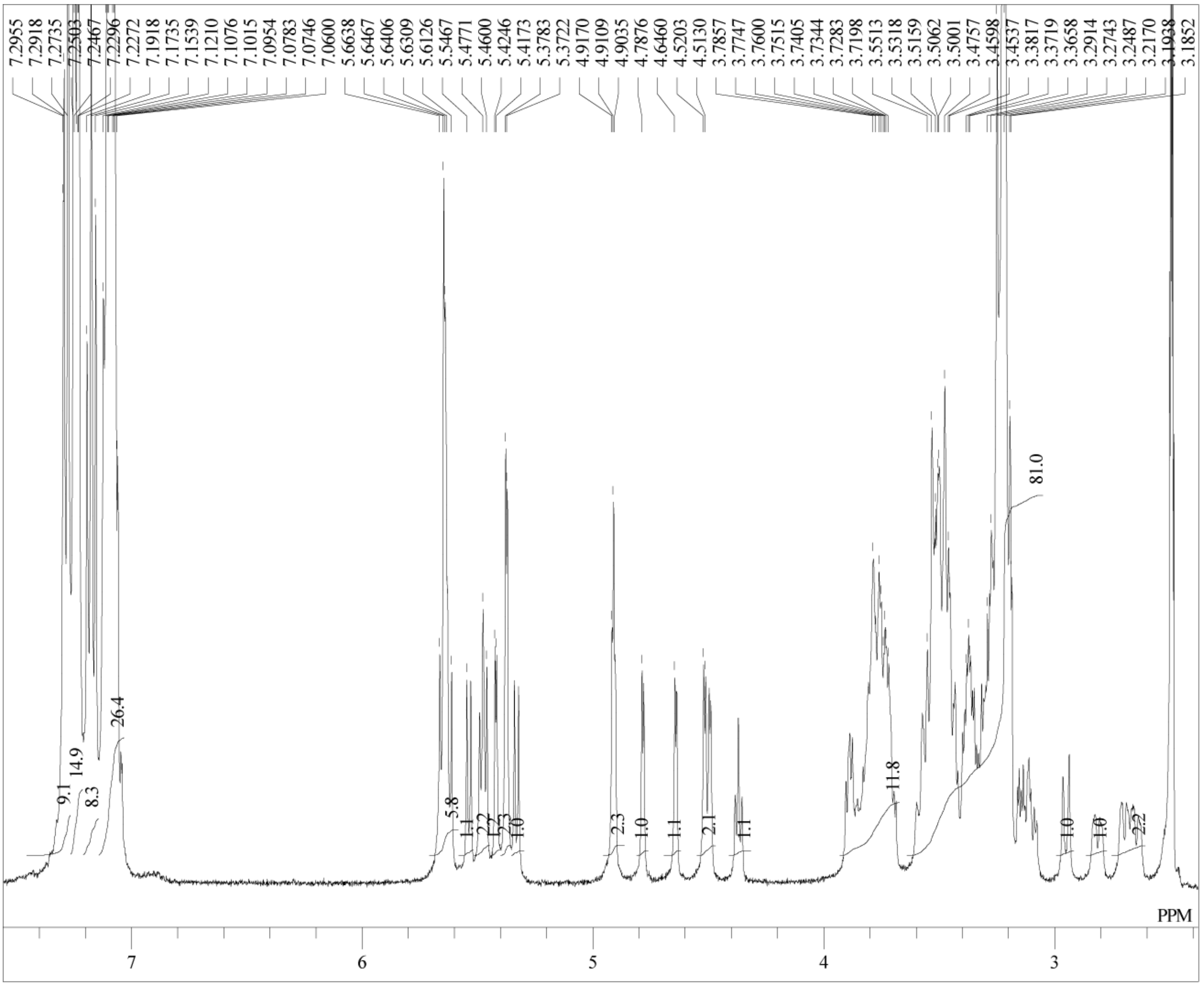

$6^A,6^B,6^E$-Tri-*O*-trityl-α-CD (ABE-isomer)

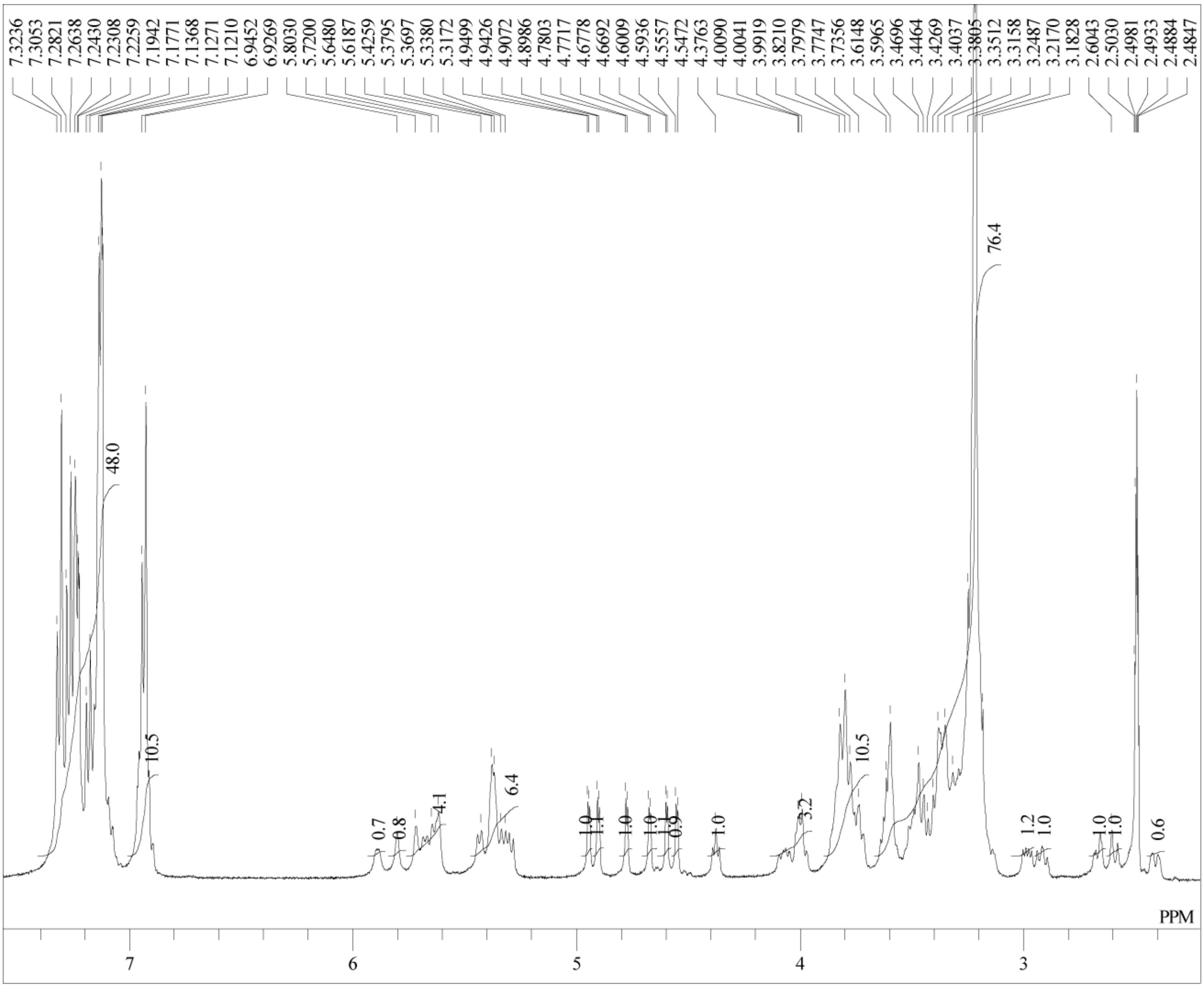

6^{A},6^{C},6^{E}-Tri-*O*-trityl-α-CD (ACE-isomer)

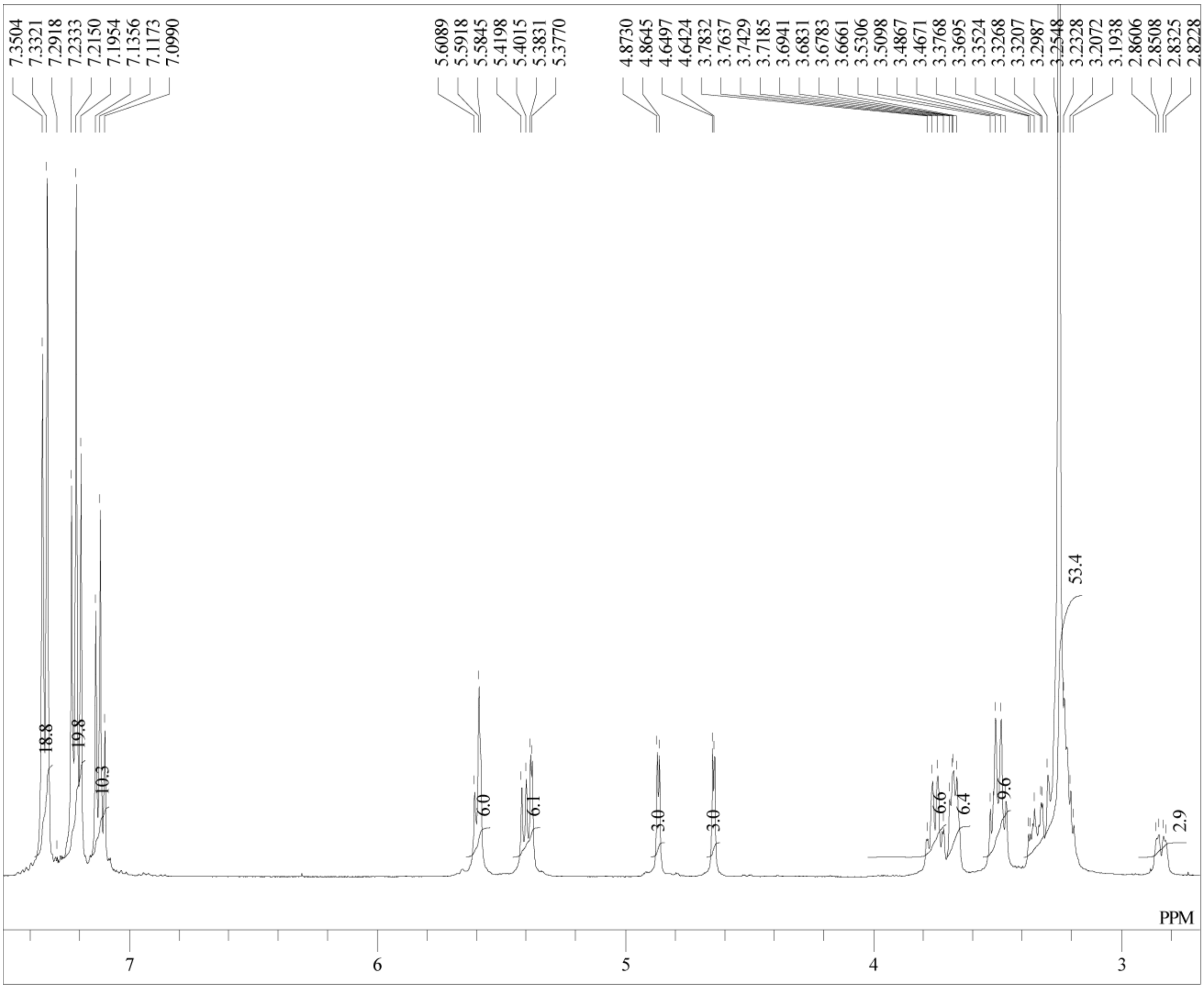

2D COSY spectrum of the AD-isomer in DMSO-d_6 at 50 °C.

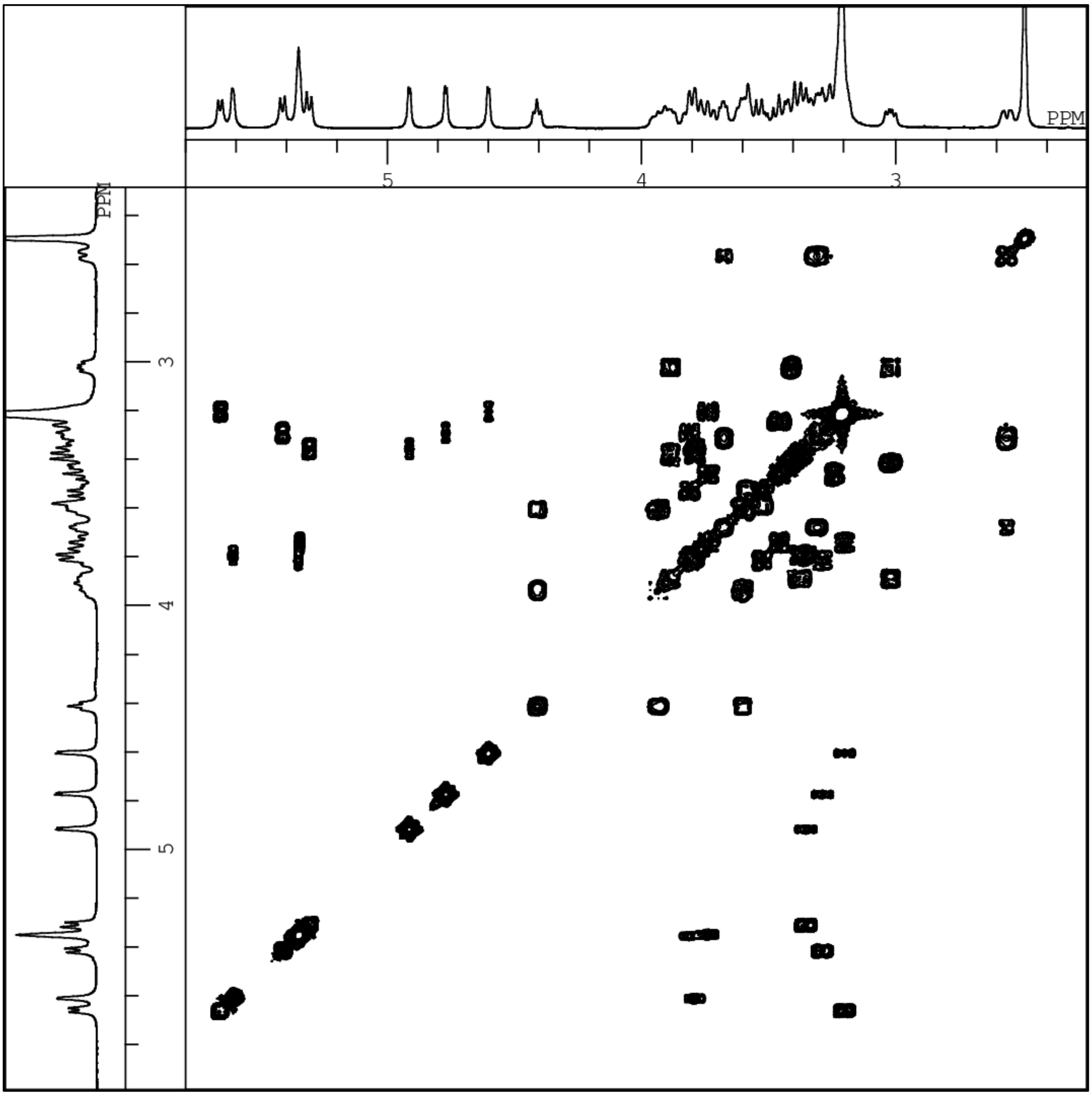

2D TOCSYspectrum of the A,D-isomer in DMSO-d_6 at 50 °C.

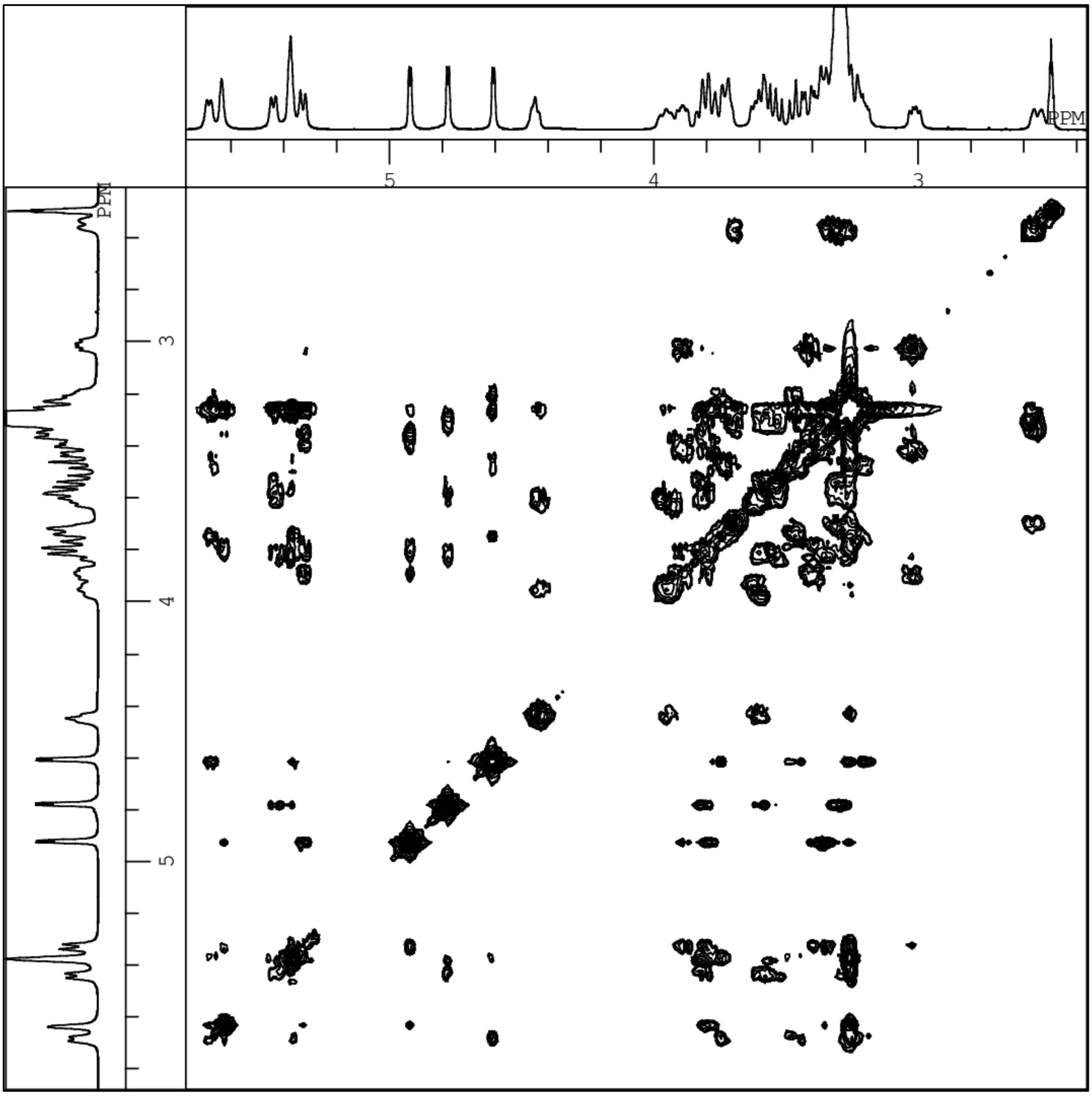

Supplement: File 1 — 1H NMR spectra of mono-, di-, and tri-O-trityl-α-CD, together 2D COSY and TOCSY spectra of the AD-isomer. [file Beilstein_J_Org_Chem-11-1530-s001.pdf]
